# Unravelling Surface Modification Strategies for Preventing Medical Device‐Induced Thrombosis

**DOI:** 10.1002/adhm.202301039

**Published:** 2023-10-05

**Authors:** Cuong Hung Luu, Nam‐Trung Nguyen, Hang Thu Ta

**Affiliations:** ^1^ School of Environment and Science Griffith University Nathan Queensland 4111 Australia; ^2^ Queensland Micro‐ and Nanotechnology Centre Griffith University Nathan Queensland 4111 Australia

**Keywords:** medical devices, neoendothelialization, surface coatings, surface modification, thrombosis

## Abstract

The use of biomaterials in implanted medical devices remains hampered by platelet adhesion and blood coagulation. Thrombus formation is a prevalent cause of failure of these blood‐contacting devices. Although systemic anticoagulant can be used to support materials and devices with poor blood compatibility, its negative effects such as an increased chance of bleeding, make materials with superior hemocompatibility extremely attractive, especially for long‐term applications. This review examines blood–surface interactions, the pathogenesis of clotting on blood‐contacting medical devices, popular surface modification techniques, mechanisms of action of anticoagulant coatings, and discusses future directions in biomaterial research for preventing thrombosis. In addition, this paper comprehensively reviews several novel methods that either entirely prevent interaction between material surfaces and blood components or regulate the reaction of the coagulation cascade, thrombocytes, and leukocytes.

## Graphical abstract

Blood‐contacting medical devices play a crucial role in contemporary healthcare but the risks of thrombosis complications due to foreign materials are a matter of concern. The figure provides the overview of critical strategies for surface modification to prevent medical device‐induced thrombosis.

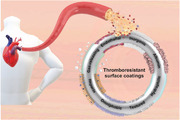

## 1 Introduction

Blood‐contacting biomedical devices are widely used in clinical remedies for various diseases regardless of short‐ and long‐term applications. For instance, casing and valves of artificial hearts, vascular grafts, stents, or catheters are often employed to treat cardiovascular diseases for a prolonged duration, while dialyzers, blood collection tubes, or tubing are applied for temporary purposes. Indwelling central venous catheters and ports are essential for venous access and medication administration in cancer patients, especially those with hematological malignancies. A prerequisite requirement is that any biomaterials in contact with patients’ whole blood or blood plasma should not form thrombus or trigger coagulation. In fact, critical care medicine routinely utilizes cardiopulmonary bypass (CPB) to maintain circulation during open‐heart surgery, which may trigger the activation of hemostatic response in clinical setting.^[^ ^1^ ^]^ Full‐dose heparin is thus required and might increase the risk of bleeding, including fatal intracranial hemorrhage and early graft malfunction.^[^ ^2^ ^]^ Otherwise, extracorporeal membrane oxygenation (ECMO), which is frequently used in patients with cardiac or respiratory failure, requires a comparatively modest dosage of heparin for anticoagulation compared to CPB. Even with ECMO assistance, hematologic issues and even brain damage remain as possible side effects.^[^ ^3^, ^4^ ^]^ Therefore, the regulation of coagulation still imposes a great challenge to those CPB and ECMO in specific and blood‐contacting medical devices as well. Although progress has been made, major problems still remain to be solved, considering the design of blood compatible materials. Several pathways to make bulk materials more blood compatible have been attempted. Examples are particular synthesis, blending, or surface coating to regulate the blood reaction. To create biomaterials that are compatible with blood, it is crucial to understand how blood components interact with foreign biomaterials and how surface features affect these interactions. From there, multiple strategies for creating hemocompatible surfaces can be developed, varying from surface passivation to functionalizing surfaces with bioactive chemicals or endothelialization.

This review revisits the coagulation cascade, the interaction between artificial surfaces and blood plasma, providing a general understanding of such events that eventually induce coagulation and thrombosis formation. The review further discusses hemocompatible coatings according to four surface modification strategies: i) inhibition of protein and cell adsorption, ii) inhibition of thrombin generation and fibrin formation, iii) inhibition of platelet aggregation and activation, and iv) endothelialization. Within the last five years, literature reviews about thromboresistant coatings have been published.^[^ ^5^, ^6^, ^7^, ^8^, ^9^, ^10^, ^11^ ^]^ However, these papers did not clearly mention how each coating material interferes and regulates the clotting cascade or indicate specific pros and cons of materials that influence the outcome of antithrombosis regulation. Therefore, the effect of material characteristics on antithrombotic activity will be emphasized and correlated with the underlying mechanisms of action in this review. Furthermore, this literature review will provide a big picture of each thromboresistant material, including advantages and limitations, outstanding achievements, and up‐to‐date studies, thereby offering an insightful understanding of the current progress of antithrombotic surface materials.

## 2 Fundamentals of Surface‐Induced Thrombosis

### 2.1 Coagulation Cascade

The blood clotting cascade is a set of actions in response to bleeding or association brought on by tissue damage or contact with artificial surfaces.^[^ ^12^, ^13^, ^14^, ^15^, ^16^, ^17^ ^]^ Each action leads to the next, which ultimately causes the formation of a blood clot (thrombosis). Thrombosis within a blood vessel can result in fatal clinical conditions such as heart attack, stroke, pulmonary embolism, etc.^[^ ^18^, ^19^, ^20^, ^21^, ^22^, ^23^, ^24^, ^25^, ^26^, ^27^, ^28^, ^29^, ^30^, ^31^, ^32^ ^]^ In particular, a number of clotting factors (**Table** **1**) start off as zymogens, which are inactive forms of proteins in blood plasma. Once activated, these serine proteases as active clotting factors accelerate the degradation of downstream protein. Principally, a clotting factor can catalyze the subsequent process once activated by its glycoprotein co‐factor. An “a” is added after the corresponding Roman number to indicate a clotting factor's activation (e.g., when activated, factor V (fV) becomes fVa). Coagulation can be split into three pathways, the extrinsic, intrinsic, and common pathways that are detailed in the following sections.^[^ ^12^ ^]^

**Table 1:** Plasma blood coagulation factors that involve in the blood clotting cascade. Reproduced (Adapted) with permission.^[^ ^12^ ^]^ Copyright 2015, Taylor & Francis, Informa.

Coagulation factors	Other name(s)	Molecular weight [kDa]	Plasma concentration [mg dL^−1^]
fI	Fibrinogen	340	200–400
fII	Prothrombin	72	12
fIII	Tissue factor, tissue thromboplastin, thromboplastin, CD142	46^a)^	—^b)^
fIV	Calcium ion	—	8.5–10.5
fV	Proaccelerin (labile factor)	330	0.4–1.4
fVII	Proconvertin (stable factor)	48–50	0.05–0.06
fVIII	Antihemophilic factor	1000–12 000	0.5–1.0
fIX	Christmas factor	57	0.4–0.5
fX	Stuart–Prower factor	57	0.7–1.2
fXI	Plasma thromboplastin antecedent	160	0.4–0.6
fXII	Hageman factor	80	1.5–4.5
fXIII	Fibrin stabilizing factor	320	1–2
Plasma prekallikrein	Fletcher factor	85 or 88	3.5–4.5
High molecular weight kininogen	Fitzgerald, Williams, or Flaujeac factor	120	8–9
Plasminogen	–	92	20

^a)^ Other document noted the value from 50–330 kDa;^[^ ^33^ ^]^;

^b)^ An integral‐membrane protein principally located on adventitial cells, thus no plasma concentration.

Tissue factor (TF), also called tissue thromboplastin (coagulation factor III or CD142), is a glycosylated transmembrane protein that expresses on perivascular cells and epithelial cells.^[^ ^12^ ^]^ The extrinsic pathway of triggering blood clotting is mainly based on TF and is thus called the tissue factor pathway. This pathway is the mechanism of normal hemostasis. In fact, there is no TF expression on the innermost endothelium layer, it only exposes and comes to contact with blood when the endothelial tissue is injured, or a blood vessel is damaged. Immediately, the clotting cascade initiates by the complexation of TF and fVIIa with the help of divalent ions (Ca^2+^). fVIIa originated from the zymogen form (fVII) that requires vitamin K to be activated. As a result, the TF‐fVIIa‐Ca^2+^ complex will trigger the common pathway which will be detailed further in Section 2.3.^[^ ^12^ ^]^

Apart from the extrinsic pathway, the intrinsic pathway only initiates when certain biomaterial surfaces come into contact with plasma in the presence of Ca^2+^, without a TF source from perivascular cells.^[^ ^9^ ^]^ It has been thus defined as the contact pathway as well. The mechanism of triggering the intrinsic pathway is emphasized by the activity of three primary zymogens, including fXII (Hageman factor), fXI, and high molecular weight kininogen (HK).^[^ ^12^ ^]^ It is noteworthy that fXII is a single polypeptide chain that deliberates in plasma, while fXI and HK exist as non‐covalent complexes (PK‐HK and XI‐HK). In the first stage, small quantities of activated factor XII (fXIIa) are produced when blood contacts an artificial surface via autoactivation.^[^ ^34^ ^]^ Then, this enzyme turns PK‐HK into kallikrein, creating a positive feedback loop in which prekallikrein (PK) and fXII are both activated in turn by kallikrein. A scant amount of fXIIa in the loop activates its downstream coagulation factor, fXI (XI‐HK), into fXIa which further provokes proteolysis of fIX to fIXa. At this point, circulating fVIII was pre‐triggered by thrombin (fIIa) combines with fIXa in the presence of Ca^2+^ to form an intrinsic tenase complex (fIXa‐fVIIIa‐Ca^2+^) which in turn completes the activation phase of the contact system by the generation of fXa from fX.^[^ ^12^ ^]^

The intrinsic and extrinsic routes, which are distinct but interconnected, consequently reciprocate to the common pathway to cause blood clotting.^[^ ^12^ ^]^ After the activation of fX as a result of either pathway, the common pathway ensues with the production of a prothrombinase via a complexation among fXa, fVa, and Ca^2+^, which starts the common route. In this case, fVa acts as fXa's receptor on the platelet membrane, which turns out to induce platelet activation and secreting prothrombin (fII). Simultaneously, prothrombin is subsequently converted to thrombin (fIIa) by the prothrombinase complex. Nevertheless, platelet aggregate formed by platelet‐fibrinogen interaction is just a premature thrombus. As it progresses, fibrinogen (fI) is cleaved by thrombin and entraps activated platelets within a network of fibrin (fIa) strands. Finally, the stabilizing factor (fXIII) is converted by thrombin into fXIIIa which again solidifies the interconnecting fibrin in the presence of Ca^2+^.^[^ ^12^ ^]^

### 2.2 Interactions of Biomaterials and Blood Plasma

Human whole blood is a complex biological fluid that contains red blood cells (RBCs), white cells, and platelets (≈45% of volume) suspended in blood plasma (≈55% of volume).^[^ ^35^ ^]^ Under ordinary physiological conditions, blood remains in contact with only the normally antithrombogenic endothelium layer which inhibits platelet adhesion, aggregation, and activation, helps prevent the activation of the coagulation cascade, and regulates hemostasis by expressing and secreting a variety of biomolecules or transmitter.^[^ ^36^ ^]^ Besides, a myriad of complex processes and interactions involving thrombocytes, the vascular endothelium, the coagulation cascade, the complement system, and the fibrinolytic system maintains a balance between thrombus generation and destruction.^[^ ^37^ ^]^ Once biomaterials come into contact and interact with one or more aforementioned components of the blood coagulation system, they may unintentionally disrupt the hemostatic balance, which enables the trigger of the intrinsic pathway and amplifies the thrombus formation.^[^ ^12^ ^]^

In contrast, artificial surfaces lack of such endothelial characteristics. Thus, they face a challenge in resisting the natural coagulation process and further lead to the failure of implanted equipment.^[^ ^38^, ^39^ ^]^ The non‐specific protein adsorption is the first event that take places immediately, following the blood‐contacting device contacting biological fluids (fouling).^[^ ^40^, ^41^, ^42^ ^]^ The process of protein adsorption is dynamic and complicated, which can be stimulated by several driving forces (hydrophobic/electrostatic interactions and van der Waals), and frequently includes the overlapping of adsorption and repulsion.^[^ ^43^ ^]^ It is influenced by both internal and external stimuli as well.^[^ ^40^ ^]^ For instance, pH, ionic strength,^[^ ^44^ ^]^ temperature,^[^ ^45^ ^]^ and concentration of plasma proteins are external stimuli of the surrounding biological milieu, whereas the physico‐chemical properties (e.g., topography, wettability, charge, etc.) of implant surfaces play the major role in protein adsorption. Besides, size and shape of proteins themselves also have an impact on the way they adsorb.^[^ ^46^ ^]^

Reports in the literature have shown that just a little quantity of protein in the plasma is enough to impact the performance of implanted devices, and the protein that has been adsorbed can create a coating that acts as a mediator layer(s) at the interface of device surface and blood components.^[^ ^47^ ^]^ Besides the mentioned zymogens, the most prevalent proteins in plasma are albumin, immunoglobulins, and fibrinogen which together make up more than half of all plasma proteins and have been identified as representatives of plasma proteins.^[^ ^40^ ^]^ With respect to surface‐induced thrombogenicity, intermediate‐sized proteins such as albumin and immunoglobulins are prone to have a higher tendency in structural transformation upon adsorption than smaller proteins.^[^ ^40^, ^46^ ^]^ In the initial stage of adsorption, small proteins predominate because they diffuse more quickly than larger ones.^[^ ^43^ ^]^ Consequently, the large proteins can competitively expel the pre‐adsorbed protein due to a larger contact area.^[^ ^48^ ^]^ As a consequence, the plasma proteins adsorption led to the conformational and/or orientational changes and conceals certain platelet‐binding sites.^[^ ^49^ ^]^ However, albumin is relatively inert to platelet adhesion and activation owing to its lack of binding sites for platelet receptors.^[^ ^50^ ^]^ Furthermore, fibrinogen has been characterized as the central protein in promoting platelet adhesion and aggregation by serving as a ligand for platelet binding (GPIIb/IIIa, GPIb, etc.).^[^ ^51^ ^]^

In the initial stage, minimal circulating platelets are activated, following by a mass of platelet activation. Many neighboring platelets are systemically activated to intensify coagulation as clotting factors released from platelet granules during the initial wave of activations. Substantial quantities of adenosine diphosphate (ADP) and adenosine triphosphate (ATP), for example, are emitted from the dense granule of thrombocytes, which can then activate nearby platelets via ADP and ATP sensitive receptors (P2Y1 and P2Y12).^[^ ^52^ ^]^ In addition, the protease activated receptors 1 and 4 are two other platelet receptors that are triggered via the thrombin generation. Additionally, it enhances the clotting cascade by triggering fV, fVIII, and fXI.^[^ ^53^ ^]^ Neutrophils also attach to the adsorbed fibrinogen via CD11b/CD18 receptor thereof.^[^ ^54^ ^]^ In addition, the surface of activated platelet could promote the adhesion of leukocytes via P‐selectin, which generates free radical substances and releases bioactive compounds (platelet activating factor, interleukins, and tumor necrosis factor, etc.).^[^ ^55^ ^]^ Similarly, the passive adhesion of erythrocytes can secrete ADP under high shear stress.^[^ ^56^ ^]^ These processes facilitate not only local platelet adhesion but also activation of activated complement components. Altogether, the fibrin mesh entraps surrounding biomolecules, including platelets, leukocytes, RBCs, plasma proteins, etc. to promote a blood clot.^[^ ^12^ ^]^

Besides the influence on the contact activation and coagulation cascade, several surfaces tenaciously trigger the activation of complement systems as well.^[^ ^9^ ^]^ In particular, the surface that pre‐adsorbs fXII and fibrinogen can spark the response from innate immune system. One of the most abundant complement components⁠—C3, is inclined to undergo structural changes upon binding to that layer, thereby leading to the activation of the alternative pathway.^[^ ^57^ ^]^ On the other hand, β‐fXIIa generated by kallikrein from contact activation also initiates the classical pathway that generates C3a and C5a.^[^ ^57^ ^]^ In general, anaphylatoxins C3a and C5a resulting from the complement activation serve as the influential chemoattractants to appeal and activate leukocytes, which eventually leads to the inflammation and advances the thrombus formation.^[^ ^39^ ^]^ Overall, with only the exposure of artificial surface to blood, a series of chaotic reactions takes place orderly or reciprocally that creates two major consequences: thrombosis formation and inflammation, which ensues the failure of blood‐contacting medical devices.^[^ ^12^, ^58^ ^]^

## 3 Several Approaches in Surface Treatment

Various surface coating techniques have been used in recent years to enhance the characteristics of material surfaces and their multifunctional uses (**Table** **2**). Depending on the substrate materials and desired optimum features, proper coating strategies could be applied specifically. In general, coating strategies can be categorized as physical and chemical methods. Physical approaches possess a physical interaction between coating films and substrates. The binding forces are various that rely upon methods and the nature of materials which comprises of all surficial properties of materials that could be considered, including surface energy, functionalities, interactions(s) between materials and coating layer. Otherwise, the chemical approaches proceed when there is a chemical reaction between coatings and surface functionalities. Some chemical modifications that have been widely used for blood‐contacting devices was summarized in **Table** **3**.

**Table 2:** Surface coating strategies for antithrombosis on blood‐contacting medical devices.

Coating	Anticoagulant mechanism	Limitations	Comments
Titanium dioxide (TiO_2_)	Reduce surface energy and work function of coating films Prevent fibrinogen from conformational change	Difficult to control anticoagulant activities due to stimuli‐dependence	Often applied on titanium‐composed substrate
Carbon‐based	Lower surface energy Expose inertness to blood components	Surface irregularities and defects Trials show that patients still need to take anticoagulant drug after implantation	Unclear overall patency Can be improved by engineering the crystalline structure and uniformity
Albumin	Inhibit further plasma proteins adsorption, especially fibrinogen	No significant antithrombogenicity	Nontoxic and able to passivate surfaces
Hydrophilic polymers	Exhibit a hydrophilic surface that reduces protein adsorption by the hydration force and steric repulsion Lessen surface energy	Several sorts of polymer cause inflammatory response Easily affected by environmental stimuli and prone to leach Highly dependent on coverage density	Hydrophilic and biocompatible Reduce protein denaturation and platelet adhesion
Zwitterion (phosphobetaine, sulfobetaine, carboxybetaine)	Increase hydrophilicity and decrease surface energy	Tend to leach and undergo oxidation degradation Limit protein adsorption but also reendothelialization	Limited neoendothelialization
Phosphorylcholine	Possess similar zwitterionic behavior with higher compatibility	Unstable coating No clear benefit of coating confirmed	Potential and applicable but require a combination of other materials
Elastin‐inspired	Express the intrinsic nature to limit platelet adhesion and aggregation	Difficult to extract and purify due to hydrophobicity Elastin‐inspired oligopeptide show promising outcomes, yet still insignificant	Potential and applicable but lack of further studies
Textured	Prevent further protein and cell adsorption, create a neointimal layer primarily composed of smooth muscle cells and fibroblasts Promote a more favorable endothelial phenotype	Difficult to manufacture and highly substrate‐dependent Bear the risk of reverse thrombosis and inflammation	Depend on a variety of factors, including the type of device, the location of the implantation, and the patient's individual physiology
Omniphobic	Repel or lubricant protein and cell interaction by fluorinated or PTFEP coating	Lack of compelling evidence in vivo Might lessen neoendothelialization	Repel all classes of liquid, specifically whole blood
Heparin	Establish heparin‐ATIII complex that disrupts thrombin function	Require high mobility and rich content of pentasaccharide sequence to catalyze antithrombin capacity	Less efficiency in infants or patients with ATIII deficiency Overcome limitations by end‐on conjugation to enhance heparin‐ATIII complex formation rate
Thrombin inhibitors	Inhibit specific factor(s) that collapse the downstream activation of contact pathway	Depend on the amount of coated thrombin inhibitor May interfere with the normal clotting process, which can increase the risk of bleeding	Improve the safety and effectiveness but still have limitations
Platelet inhibitors	Disable directly/indirectly platelet activation via different pathways	Chemical conjugation of coatings may affect the efficiency	Improve the safety and effectiveness but still have limitations
NO, H_2_S	Simulate healthy vasculature by releasing gaseous transmitter Inactivate platelets, promote endothelialization, inhibit inflammatory and SMCs proliferation	Release only a certain amount depending on incorporated precursor concentration Risk of heavy metal ion leakage	Potential and multifunctional Need further investigations for sustained release and therapeutic window of safety
Neoendothelialization	Simulate healthy vasculature by coating ECs	Require allogenic ECs Risk of bacterial infection The process takes time, which may not be practical for some applications	Promising approach to prevent thrombosis in blood‐contacting devices Remaining limitations need to be taken into consideration

PTFEP—poly(bis(2,2,2‐trifluoroethoxy)phosphazene), SMC—smooth muscle cells, ECs—endothelial cells.

**Table 3:** A summary of chemical surface modifications on materials applied for blood‐contacting devices.

Methods	Technical features	Advantages	Disadvantages
Electroplating	Transformation of ionic metal into nonionic coating by electrons from current	Uncomplicated design Applicable for complex workpieces or substrates Easy to control composition	Difficult to optimize uniformity Limited coating (only used for metallic coatings)
Micro‐arc oxidation	Conversion of bare metal into ceramic oxide coating by electric energy	Straightforward design Applicable for complex workpieces or substrates	Uncontrollable crystalline structure and uniformity
Chemical vapor deposition	Deposition at determined conditions to form coating film via chemical reaction	Easy to control composition and characteristics Applicable for complex workpieces or substrates Enable to produce crystalline or amorphous films	Low process rate High demanding in vapor conditions (precursor, flow rate, etc.) High pollution due to by‐products
Other vapor depositions	Deposition of materials evaporated by various kinds of energy sources	Enable to optimize uniformity and density Easy to control composition	High defection High demanding in vapor conditions
Sol–gel	Preparation of oxide layer(s) onto dipping surfaces	High uniformity of coating surfaces Suitable for diverse sizes of wafers Require low temperature and simple operation	Excessive cost Prolonged process
Grafting	Generation of covalent/ionic bonds via chemical reactions between functionalities of surfaces and coatings	Stable, reliable Suitable for varied sizes and shapes of substrates Applicable for most organic materials	Material‐dependent Difficult to optimize uniformity and density due to steric hindrance

For metal‐based substrates, popular technology is electrodeposition surface coating, such as plating (galvanizing), plasma electrolytic oxidation or micro‐arc oxidation, etc. These techniques are basically established from the well‐known Faraday's law that have the mass of metallic substance which is either deposited or undergone anodic oxidation. For example, micro‐arc oxidation was also used to fabricate protective metal oxide layers on the surface of valve metals and alloys which displays good adherence to the substrate with high hardness, high resistance to corrosion and wear.^[^ ^59^, ^60^ ^]^ However, the electrolyte/substrate interface plasma discharge will result in microdefects and a porous surface, reducing corrosion resistance and failing to provide long‐term corrosion protection.^[^ ^61^ ^]^ The leakage of metallic ions could result in contaminating the bloodstream and cause undesired side effects to implantation. Hence, there is a need for sealing techniques to further enhance the anticorrosion capabilities of substrates. These techniques include adding corrosive inhibitors and sol‐gel sealing via dip coating.^[^ ^62^ ^]^ Furthermore, chemical vapor deposition (CVD) and atomic layer deposition (ALD) is a chemical method, in which a substrate is exposed to one or more volatile precursors. The precursors then react or break down on the surface of the substrate and form the desired thin film deposit. Carbon‐based coatings are often employed by this method; however, this method is costly and shape substrate dependent. Like CVD and ALD, several advanced deposition methods have been exploited as well, including ion beam enhanced/aided deposition, plasma immersion ion implantation and deposition, or magnetron sputtering deposition, etc. These strategies have shown potential in uniformly depositing high‐aspect‐ratio assemblies on substrates or producing organic‐inorganic hybrid layers with controlled thickness.^[^ ^62^ ^]^ However, these approaches require strict contamination control, partial pressure, or further treatments (thermal, plasma) to adjust the coating performance at the atomic or molecular level.^[^ ^63^ ^]^ Besides, it is challenging and costly to manufacture a homogeneous coating film over a substrate with intricate forms or geometry, most studies were therefore conducted on plain surfaces.

Apart from above methods, sol–gel coating is one of the most prevalent chemical methods that was widely used to introduce oxide layers. A benefit of sol–gel dip coating is the low processing temperature. Moreover, sol–gel method can compensate for the drawback of physical deposition methods, as it does not depend on substrate form and can acquire good control over surface properties like composition, thickness, and topography.

Last but not least, grafting is the most popular coating technique that could be used for a wide range of materials, from polymers (hydrogels) to bioactive molecules (proteins, peptides, medicine). Grafting is the process, where chemical groups and functionalities are ionically or covalently attached to the surface. The four dominant methods are self‐assembled monolayer,^[^ ^64^ ^]^ layer‐by‐layer assembly,^[^ ^65^ ^]^ plasma treatment,^[^ ^66^ ^]^ and mussel‐inspired polydopamine coating.^[^ ^67^ ^]^ Regarding to polymer grafting, there are two primary techniques for conjugating covalently bonded polymers: “side‐on grafting,” in which polymers are immobilized on the surface via active sites along their backbones, and “end‐on grafting,” in which initiators have been conjugated to the surface and the polymerization proceeds in situ.^[^ ^68^ ^]^ The disadvantage of “side‐on grafting” is, however, the constrained coating density brought on by steric hindrance during the immobilization process, which permits the adsorption of smaller proteins among the immobilized macromolecules.^[^ ^69^ ^]^ Therefore, “end‐on grafting” is now the favorable but more expensive and complex option due to the higher coating density that is attained.

## 4 Surface Coating to Prevent Blood Clotting

### 4.1 Inhibition of Protein and Cell Adsorption

**Figure 1:**
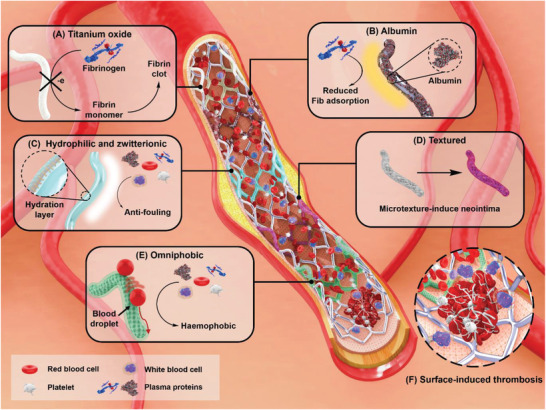
Illustrative depiction of various antithrombotic coatings, underlying mechanisms of action of A) titanium oxide, B) albumin, C) hydrophilic polymers and zwitterions, D) textured surfaces, E) omniphobic surfaces toward the inhibition of protein and cell adsorption. The interference of artificial stent could cause a F) thrombosis via the intrinsic pathway and inflammatory response via the complement activation.

Anti‐biofouling layers play a pivotal role in surface coating for inhibition of the thrombosis formation, which mainly suppresses the first action of the interaction between the synthetic surface and blood—the adsorption of circulating proteins and cells. A significant amount of research has been done to create anti‐protein adsorption surfaces because plasma proteins are crucial in controlling the blood compatibility of implants.^[^ ^9^ ^]^ This has reduced platelet adhesion, controlled blood coagulation, and enhanced blood compatibility of the implants.^[^ ^70^, ^71^ ^]^ In particular, antifouling surfaces often focus on reducing the intermolecular forces between extracellular biomolecules and the surface to prevent adhesion of proteins and cells or to facilitate the deliberation of them under shear stresses. Several thromboresistant coating strategies were illustrated in **Figure** **1**, these coatings underlie the inhibition of protein and cell attachment to prevent surface‐induced thrombosis. Also, critical research about anti‐fouling coating materials were summarized in **Table** **4**.

**Table 4:** Critical studies in surface coatings inhibiting protein and cell adsorption for antithrombogenicity.

Substrate/device	Coating	Coating technique	Critical properties		Key results	Ref.
LTIC	TiO*_x_*	Ion beam enhanced deposition	—	—	Reduced the aggregation of platelets, formation of pseudopodia and globulicidal effect Ti^2+^ and Ti^3+^ in titanium oxide films lessened function Albumin/fibrinogen ratio implied the hemocompatibility of TiO_2_ No obvious fibrin formation and RBCs maintained normal shape in vivo	[232]
Ti	Anatase‐ and/or rutile‐phase TiO_2_	Heat and/or H_2_O_2_ treatment, and sol–gel dip‐coating	*R*_a_ *θ*_c_	0.65–0.8 µm (heat and/or H_2_O_2_ treated) <60° (H_2_O_2_ treated) >60° (no H_2_O_2_ treated)	The sol–gel coatings highly showed blood compatibility regardless anatase or rutile phase H_2_O_2_‐treated and heated (550 °C) Ti surface exhibited the best blood compatibility	[233]
Ultrafine‐grained pure titanium	Anatase‐ and/or rutile‐phase TiO_2_	Micro‐arc oxidation	*t* *R*_a_ θ_c_	2.63–14.02 µm 1.11–3.84 µm 13.2 ± 2.73° (lowest) 22.9 ± 3.30° (highest)	Reduced the hemolysis rate, the amount of platelet adhesion, and the degree of deformation Extended the dynamic coagulation time	[234]
Ti	TiO*_x_*	Oxygen plasma immersion ion implantation treatment	*t* *R*_a_ *θ*_c_	120 nm (low oxygen) 170 nm (high oxygen) 181 ± 37 nm (low oxygen) 176 ± 28 nm (high oxygen) 51 ± 4° (low oxygen) 52 ± 2° (high oxygen)	Rutile phase promoted a higher amount of Blood clotting and platelet activation Blood response was mainly owing to the surface oxide component, rather than surface roughness or hydrophilicity	[235]
Ti	TNTs	Anodic oxidation and heat treatment	*d* *t* *θ*_c_ BSA FIB	≈30, 50, 70, 90 nm ≈5, 7, 15, 22 µm <15° (unannealed) <5° (annealed) ≈400–500 µg cm^−2^ (unannealed) ≈500–600 µg cm^−2^ (annealed) ≈100–150 µg cm^−2^ (unannealed) ≈50–75 µg cm^−2^ (annealed)	Surface wettability, protein adsorption, blood compatibility, and ECs adhesion and proliferation impacted by the diameter, length, and crystal structure of the nanotube arrays	[63]
Ti, Ti−6Al−4V	TiO_2_	Hydrothermal treatment	*θ*_c_ BSA FIB	<47 ≈60–90 µg cm^−2^ ≈4–14 µg cm^−2^	Small‐sized dense porous structure revealed higher hemocompatible than granular surfaces	[94]
Mg–Zn	Amorphous TiO_2_	Magnetron sputtering	*t* *R*_a_ *θ*_c_	400 nm 51 nm 79.6°	Minimized the hemolysis rate compared to the bare surface No significant anti‐platelet adhesion	[236]
Ti	TNTs	Anodization with or without O_2_ plasma treatment	ø *R*_a_ *θ*_c_	15, 50, 100 nm (jointly increased with potential used) 10.1, 15.5, 25.9 nm <5° (as prepared and plasma exposure) >80 (atmosphere exposure)	O_2_ plasma treatment reduced the platelet adhesion Plasma‐treated TNTs also provided the appropriate environment for ECs attachment and growth but prohibited SMCs adhesion	[237]
Ti	TNTs	Annealed TNTs prepared by anodic oxidation and further coated with DA‐Zn^2+^	*θ_c_* BSA FIB	32.5° (TNTs) <18.7° (annealed and Zn^2+^ chelated TNTs) ≈5–9 µg cm^−2^ ≈2–2.5 µg cm^−2^	Increased the ratio of BSA to fibrinogen followed by the increasing feed of Zn^2+^ chelating Reduced the platelet activation significantly Encouraged the growth of the ECs with Zn^2+^ release but also increased the hemolysis rate	[238]
Titan2	Ti─N─O	Plasma enhanced vapor deposition	—	—	Showed a non‐inferiority (a rate of cumulative MACE) at 12‐month follow‐up compared to everolimus‐eluting stents in randomized BASE‐ACS trial	[239]
Titan2	Ti─N─O	Plasma enhanced vapor deposition	—	—	Revealed better clinical outcome compared to paclitaxel‐eluting stents at 5‐year follow‐up in randomized TITAX‐AMI trial	[240]
PVC	Al_2_O_3_	Thermal or plasma‐enhanced atomic layer deposition	*R*_a_ *θ*_c_	65.7 ± 7.9 nm (thermal ALD) 65.4 ± 11.8 nm (plasma‐enhanced ALD) 122.7 ± 3.4° (thermal ALD) 81.0 ± 2.9° (plasma‐enhanced ALD)	A smaller amount of prothrombin was absorbed on thermal ALD Al_2_O_3_ No activation of blood cells A thin albumin layer was absorbed on thermal ALD Al_2_O_3_ substrates due to the residual methyl groups	[61]
PET/arterial graft	Albumin	Albumin covalently cross‐linked by glutaraldehyde	—	—	Expressed the non‐immunogenicity in vivo in mice Decreased the platelet adhesion and activation in dog	[143]
PVC	PDA and PDA/AgNPs	Co‐deposition of dopamine and Ag^+^	*θ*_c_ FIB	<5° 38 ng cm^−2^ (PDA) 65 ng cm^−2^ (PDA/Ag)	Formed the superhydrophilic surface shielded non‐specific proteins and maintained the conformational structure Prevented platelets adhesion and activation, reduced thrombosis formation in vivo Inhibited the adhesion, activation, and growth of macrophages	[241]
Tygon CPB tubing	PDMAA	PDMAA coated on tubing surface by UV initiator	*t* *θ*_c_	10 µm <40°	Experienced lower fibrin coverage and friction coefficient in PVC tubing Decreased blood clotting in tubing loops and occlusion time in porcine model	[242]
Various surfaces	Silsesquioxane/PMEA	Silsesquioxane and PMEA reacted via thiol‐initiated polymerization	*R*_a_	<0.972 ± 0.048 nm	Suppressed the human platelet adhesion Promoted cell adhesion and expansion of HUVECs onto different coated surfaces in vitro	[243]
Glass	Biodegradable PC copolymer	Ultrasonic spraying	*t* *θ*_c_ BSA	2.12 ± 0.20 µm <67.0 ± 0.37° <6 µg cm^−2^	Expressed satisfactory blood compatibility, anti‐adhesion properties and biodegradability Sustained rapamycin release profile in drug‐eluting stent Suppressed neointimal hyperplasia in a porcine artery injury model in vivo	[244]
Ti	PSBMA‐ or PCBMA‐grafted TiO_2_ nanotubes	TiO_2_ nanotubes prepared by electrochemical anodization on Ti sheets and grafted by PSBMA or PCBMA	*t* BSA FIB	≈13–18 nm <1.5 µg <4.5 µg	Reduced protein adsorbed dramatically, and suppressed the effects of the surface topography Reduced the adhesion and activation of the platelets	[245]
Glass	PC and carboxylic groups	Copolymers bearing phosphorylcholine and carboxylic groups	*θ*_c_ BSA	<20° ≈750 µg cm^−2^ (control) ≈100 µg cm^−2^ (phosphorylcholine coating)	Exhibited outstanding properties in resistance of protein adsorption, platelet adhesion, and blood coagulation Suppress thrombus formation in whole blood for more than 24 h	[88]
Zn−1Mg	Zwitterionic PC chitosan	Zwitterionic PC chitosan coated on silane pre‐modified surface	*R*_a_ θ*_c_*	684.7 ± 12 nm 45.3 ± 1.56°	Exhibited minimal hemolysis ratio below 0.2% and reduced platelets and deformed RBCs adhered on the surface Promoted attachment and proliferation of HUVECs	[246]
Glass, PLA	PC and quaternary ammonium groups	Dip‐coating	*t* *θ*_c_	<350 ± 83 nm <28.41 ± 9.81°	Lowered platelet activation and adherence relative to the glass or PLA samples Observed no hemolysis Indicated effective antithrombotic effects in two hours of blood circulation ex vivo Showed no cytotoxicity to HUVECs	[247]
PET	Dihydrolipoic acid‐modified sulfobetaine‐derived starch hydrogel	Hydrogel covalently bonded to PDA‐deposited PET surface	*t* *R*_a_ *θ*_c_ Proteins	<28.4 nm <19.2 nm <21.5° >0.8 OD_562 nm_ (control) <0.15 OD_562 nm_ (hydrogel coatings)	Repelled the protein adsorption, cell, and platelet adhesion in vitro Reduced inflammation response in vivo Promoted the adhesion, proliferation, and migration of HUVECs by REDV peptide functionalization	[188]
Various surfaces	Tethered‐liquid perfluorocarbon	Plasma activation to introduced tethered perfluorocarbon and immersed in a liquid silane solution	*t* Proteins	3.4 ± 1.0 nm >2 µg cm^−2^ (control) <1 µg cm^−2^ (hydrogel coatings)	Repelled whole blood Reduced adhesion and activation of blood components Suppressed thrombosis formation under flow in vitro Reduced thrombosis in vivo	[227]
Ti	TiO_2_ nanoflowers or TNTs	Hydrothermal treatment or anodic oxidation then fluorinated or PEGylated	*R*_a_ *θ*_c_	4.6 ± 0.2 µm (nanoflowers) 1.5 ± 0.2 µm (nanotubes) <20° (PEGylated nanoflowers and nanotubes) >159° (fluorinated nanoflowers and nanotubes)	Repelled whole blood Decreased platelet adhesion and activation in fluorinated samples compared to PEGylated and non‐textured samples	[224]
Glass	PTFEP‐coated Al_2_O_3_ nanowires	Al_2_O_3_ nanowires synthesized by CVD and deposited with PTFEP	*θ*_c_	163 ± 1°	Prevented platelet attachment and activation by the smaller contact area and non‐wetting nature Reduced thrombus formation and bacterial adhesion	[223]
Ti	PTES	Laser and chemical‐treated Ti then fluorinated by PTES	*θ*_c_	165.2° (water) 154.8° (plasma) 152.1° (whole blood)	Showed excellent repellence to water, plasma, whole blood, and only a small amount of the proteins adhered	[225]

BSA—bovine serum albumin, FIB—fibrinogen, PDMAA—poly(*N*,*N*‐dimethylacrylamide), PSBMA—poly(sulfobetaine methacrylate), PCBMA—poly(carboxybetaine methacrylate), PTES—1H, −1H, −2H, −2H‐perfluorooctyl‐triethoxysilane.

#### 4.1.1 Inorganic Coatings

##### Titanium‐Based Coatings

Titanium and its alloys are one of the most used biomaterials that have emerged in various fields of technology and application, such as medicine, cosmetics, bone, and dental implants. In fact, the well‐known biocompatibility of titanium is accompanied to the native titanium oxides film on its surface. On the other hand, titanium oxides have shown their excellent hemocompatibility as well as various fabrication techniques, they thus have been suggested as coatings for blood‐contacting implants.

Regardless of the alloy composition, the thickness of the oxide layer was considered the initial factor in the impact on surface hemocompatibility. In fact, the naturally established oxide layer often has plenty of defects and is just 1.5–10 nm thick. As mentioned, this layer barely possesses the antithrombogenic property.^[^ ^72^ ^]^ Therefore, Sunny et al. conducted initial attempts to demonstrate that the thickness of the titanium layers affected protein adsorption.^[^ ^73^ ^]^ Particularly, mole ratios of the adsorbed fibrinogen and albumin decreased from 1.44 to 0.96 when titanium thickness increased from 26.6 to 128.6 nm, respectively. Likewise, Huang et al. established a natural logarithmic relationship between oxide thickness and clotting time, the clotting time increased 1.2 times when the oxidized film thickness increased from 40 to 350 nm.^[^ ^74^ ^]^ Thus, oxide thickness affects antithrombosis properties of the coating; however, the effect is not significant, and the underlying mechanism is still unclear.

Besides thickness, topography or basically surface roughness is another crucial factor in the design of biomaterials because it has a considerable influence on protein adsorption at the nanoscale.^[^ ^75^ ^]^ As a rule of thumb, the roughness modifies the materials' wettability as well as the specific surface area that is accessible for interacting with proteins and cells.^[^ ^76^ ^]^ For instance, adsorption proceeds more quickly on surfaces with higher root‐mean‐square roughness, resulting in a thicker protein layer at saturation. This fact confirms the increasing amount of accessible adsorption sites mostly controls bovine serum albumin (BSA) adsorption on titanium oxide surfaces.^[^ ^77^ ^]^ It is generally established that blood‐contacting surfaces with exceptionally smooth surface⁠—average roughness (*R*_a_) < 50 nm, would be preferential to prevent blood platelets adherence and thrombogenesis.^[^ ^78^ ^]^ On the other hand, micro‐ and nano‐roughness is perceptible to cells, and has an impact on their ability to adhere, proliferate, and grow.^[^ ^79^ ^]^ As reported, a vascular stent efficiency should be dependent on low thrombosis formation and quick endothelialization.^[^ ^80^ ^]^ Hence, further research is required to determine if endothelium can develop on micro‐ or nano‐rough surfaces. Zhou et al. studied the biocompatibility of Tca‐8113 epithelial‐like cells on amorphous TiO_2_ film with different surface roughness in nanometer scale. The results showed that neither smooth and hydrophobic nor rough and hydrophilic surfaces preferred to cell attachment in the first 6 h. Interestingly, cell spreading kept showing a notable influence by the surface texture after 16‐h culturing, which was greater on the rough surface than the smooth surface, the cell was fully spreading with remarkable density.^[^ ^81^ ^]^ However, to some extent, roughness could govern the biofouling from plasma proteins and cells, because it associates with the surface area implement and expose a higher density of binding sites. Indeed, fibrinogen with an elongated shape also revealed a higher affinity to rough surfaces compared to nearly globular albumin.^[^ ^72^ ^]^

The transport of electrons from the inactive state of fibrinogen to the surface of the artificial biomaterial relates to the development of thrombus. Surface‐adsorbed fibrinogen that has undergone oxidation and converted to fibrin monomer will swiftly cross‐link to create an irreversible thrombus.^[^ ^82^ ^]^ The inhibitory effect of TiO_2_ was first reported by Huang et al., in which TiO_2_ possesses an intrinsic n‐type semiconducting properties and has higher dielectric constant compared to fibrinogen.^[^ ^83^ ^]^ Principally, with a 1.8 eV gap, fibrinogen also exhibited the properties of a semiconductor, which was located within the forbidden band gap of TiO_2_ of 3.2 eV, thereby impeding the electron transfer from fibrinogen to TiO_2_ film, avoiding the fibrinogen denaturation, and in turn reducing the thrombus formation.^[^ ^84^ ^]^ The integration of intermediate Ti^2+^ and Ti^3+^ oxidation states in bulk coatings produced lower thrombogenicity. According to Chen et al., Ta^5+^‐doped titanium oxide films curtailed fibrin adsorption and conformational denaturation, which explained by the increment in the Fermi level of the oxide coatings.^[^ ^85^ ^]^ Moreover, La_2_O_3_‐doped TiO_2_ also resulted in the same outcome with unknown underlying mechanism. In parallel, phosphorus ion embedded TiO_2_ thin film also asserted the enhanced semi‐conducting properties, which are further enhanced by the hemocompatibility nature of phosphorus‐incorporating biomolecules such as phospholipids,^[^ ^86^ ^]^ phosphazenes,^[^ ^87^ ^]^ phosphorylcholine.^[^ ^88^ ^]^ However, the immobilization of Ca^2+^ and Ag^+^ could facilitate the platelet adhesion and activation, which could be attributed to the raise of surface energy.^[^ ^89^ ^]^ Furthermore, isoelectric point of TiO_2_ is 6.25 that will induce negatively charged when immersed in blood (pH 7.4), preventing blood components having negative charges, such as blood platelets, from adhering to the surface and facilitating more problematic blood clotting.^[^ ^90^ ^]^ Despite that, surface charges seemed to contribute the least instead of the composition, wettability, nano‐roughness, and exposed surface area.^[^ ^91^ ^]^

Aside from the aforementioned factors, crystal structure and surface energy also affect the degree of protein adsorption. Compared to anatase and amorphous TiO_2_, rutile is the most thermodynamically stable phase, which is more tightly packed with high blood compatibility.^[^ ^92^ ^]^ However, the rutile‐dominated samples with high crystallinity displayed the shortest clotting times in the experiment conducted by Tsyganov et al.^[^ ^93^ ^]^ This could be concluded that crystal structure simultaneously plays a minor role compared to other stimuli. Depending on the proportion of crystalline structures, the surface energy follows the trend: rutile > anatase/brookite > amorphous, which arbitrarily contributes to the proteins and cells adsorption. For instance, the well‐crystallized TiO_2_ film dominated by the rutile structure results in a small‐sized dense porous structure that can hinder the protein adsorption‐induced cell adhesion (platelet and leukocytes).^[^ ^94^ ^]^ Otherwise, the dielectric constant of TiO_2_ film with a higher content of rutile state, leading to a higher quantity of fibrinogen adsorption.^[^ ^95^ ^]^

**Figure 2:**
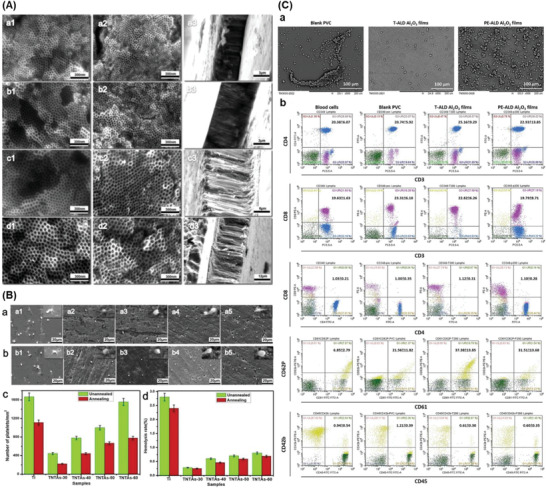
Titanium dioxide nanotubes coated surfaces. A) Scanning electron microscopy images of TNTs on Ti surface: a–d) the TNTs anodized at 30–60 V; 1,2) the non‐ and annealing heating, and 3) the cross‐sectional morphologies of (a1)–(d1). B) The SEM images of platelet adhered to a) nonannealed surfaces and b) annealed surfaces (a1) Ti; a2) TNTs‐30; a3) TNTs‐40; a4) TNTs‐50; a5) TNTs‐60; b1) Ti‐A; b2) TNTs‐30A; b3) TNTs‐40A; b4) TNTs‐50A; b5) TNTs‐60A), and c,d) the corresponding number of adhered platelets on different substrates. Reproduced with permission.^[^ ^63^ ^]^ Copyright 2019, Elsevier. C) Aluminum oxide was coated on PVC surfaces. a) Red blood cells adhesion on blank PVC, thermal atomic layer deposition (T‐ALD) and plasma‐enhanced ALD (PE‐ALD) Al_2_O_3_ films after 1 h incubating with freshly drawn human whole blood. b) Activation of blood cells evaluated by a fluorescence‐activated cell sorter, as evident in CD3^+^/CD4^+^/CD8^+^, CD61^+^/CD62P^+^, and CD45^+^/CD42b^+^ populations. Reproduced with permission.^[^ ^61^ ^]^ Copyright 2022, Wiley Periodicals LLC.

In addition, another emerging TiO_2_ structure⁠—titanium dioxide nanotubes (TNTs) feature a high specific surface area and adjustable nanotube diameter as well as length. This structure can offer a great platform for functionalization and subsequent attachment and proliferation of cells on the surface for improved hemocompatibility. Basically, the TNTs reveal similar characteristics as bulk TiO_2_ whose crystal structure is highly impactful on coating performance. The water contact angles (WCAs) of all annealed samples are below 5° regardless dimensions of tubular TNTs. As can be seen in **Figure** **2**A,B, the selective adsorption of fibrinogen may be slightly increased as nanotube diameter decreases, but it may also improve BSA adsorption to some extent that correlates to less platelet adhesion and hemolysis rate, thereby improving the blood compatibility and endothelial cell adhesion and proliferation as well.^[^ ^63^ ^]^ One advantage of TNTs is the ability to be introduced further treatments, for example, plasma treatment, hemocompatible factors doping^[^ ^96^ ^]^ or coating.^[^ ^97^, ^98^ ^]^

Aside from titanium dioxide coatings, titanium nitride (TiN) has long been used as a coating on medical devices such as heart valves, heart assist devices, and heart pumps. TiN surfaces considerably lower the likelihood of thrombus formation, much like TiO_2_ coatings, however they are less wear resistant.^[^ ^99^ ^]^ Therefore, titanium‐nitride‐oxide coating was developed that could also enhance the surface hardness and exhibit favorable blood compatibility. The coatings can be created using energetic nitriding, physical or chemical vapor deposition, and film deposition.^[^ ^100^, ^101^ ^]^ In terms of antithrombotic mechanism, TiN layer reduces the surface energy, limiting the blood component interactions. In addition to titanium oxide, TiN reveals its semiconducting properties that could further inhibit the oxidation of fibrinogen into fibrin. In preclinical studies, titanium‐nitride‐oxide coating on stents have confirmed their blood compatibility by inhibiting platelet and fibrin deposition, and lessening neointimal growth. In nonrandomized and randomized trials, titanium‐nitride‐oxide‐coated stents had an adequate safety record. However, they are inferior to second‐generation drug‐eluting stents for angiographic results.^[^ ^102^, ^103^ ^]^

##### Other Metal–Based Coatings

Due to its alleged biocompatibility, gold is employed widely for biomedical applications^[^ ^104^ ^]^ and is preferred in many medical implants without the exception of antithrombotic coatings.^[^ ^105^ ^]^ The biocompatibility of stents was supposed to be improved by coating 316L stainless steel with gold, and experimental evidence from animal studies supports this claim. Particularly with regard to thrombogenicity, gold‐coated stents caused less intima proliferation than stainless steel in dogs.^[^ ^106^ ^]^ In randomized research, the implantation of gold‐coated stents, however, was associated with a higher risk of restenosis in the first year following stenting, comparing them with uncoated stainless steel stents in patients with coronary artery disease.^[^ ^107^ ^]^ The effect might be brought on by various gold plating methods. Later, Edelman et al. demonstrated that the degree of intima proliferation in a pig model is essentially determined by the processing of the gold coating which affects the topographic characteristic of the gold layers.^[^ ^108^ ^]^

Similar to titanium oxide, aluminum oxide (Al_2_O_3_), which has excellent chemical and thermal stability, has been utilized in a number of medical equipment such as ceramic artificial joint prosthesis, fillers for dentures, and the pivot bearings in centrifugal blood pumps. Nanoporous alumina membranes were studied to improve hemodialysis. In other study, alumina thin films were deposited via thermal ALD or plasma‐enhanced ALD on poly(vinyl chloride) (PVC) circuit tubing using for CPB.^[^ ^61^ ^]^ Blood cell activation was not a result of thermal ALD or plasma‐enhanced ALD Al_2_O_3_ films, as shown by populations of CD3^+^/CD4^+^/CD8^+^, CD61^+^/CD62P^+^, and CD45^+^/CD42b^+^ cells (Figure 2C). With the relatively high lipophilicity, thermal ALD Al_2_O_3_ samples displayed a thin albuminated layer that lessened the adsorption of prothrombin. Although the anticoagulant mechanism has not been well‐established, Wang et al. suggested that the methyl (CH_3_) groups on alumina film could contribute to the enhanced anticoagulation.^[^ ^61^ ^]^ Nevertheless, it is highly dependent on the coating methods and cannot underlie the similar performance among all Al_2_O_3_ coatings. On the other hand, porous nanostructure can be generated via a specific production process which can be employed to load anticoagulant or antiplatelet drug by spraying or dipping it into a solution of the chosen drug.

#### 4.1.2 Carbon‐Based Coatings

##### Pyrolytic Carbon Coatings

Pyrolytic carbon (PyC) coating early adapted the idea of carbon‐coated surfaces to a contemporary strategy. In general, PyC has been widely used for coating and structural material on vascular grafts, stents, and mechanical heart valves due to its excellent biocompatibility, cost‐efficiency, and ease in fabrication.^[^ ^109^ ^]^ It is often manufactured by CVD method and has the graphene‐like assembled structure. However, there is an existence of covalent in lieu of van der Waal bonds between its parallel graphene lattices.^[^ ^81^ ^]^ Principally, hydrocarbon resources such as methane are decomposed at determined temperature that allows the crystallization of highly ordered sp^2^ graphitic layers. For the well‐organized structure of PyC, the coated surfaces have low surface energy, thereby eliminating the adsorption of proteins and cells.

In contradictory to other inorganic materials, PyC‐coated protheses was well‐established and investigated in animal models and clinical trials. The early results elucidated a promising patency rate of carbon‐coated surfaces owing to the significant decrease in platelet adherence and spreading.^[^ ^110^ ^]^ In particular, Arabi et al. documented an excellent hemocompatibility of graphite coated polyester vascular grafts implanted for 15 months in sheep.^[^ ^111^ ^]^ In the preliminary stages of clinical trials, Sick et al. compared Carbofilm‐coated stents (commercial thin film made of PyC) with pure high‐grade stainless‐steel stents. However, there was no advantage of the coated stents compared to the non‐coated ones.^[^ ^112^ ^]^ Similarly, clinical studies on humans have revealed a disappointing outcome that the long‐term effectiveness of carbon‐coated coronary stents and vascular grafts does not maintain high efficiency compared to that of uncoated grafts.^[^ ^113^ ^]^ Researchers found no difference in diameter stenosis at follow‐up between bare stainless steel MAC stents and carbon‐coated MAC stents in the Prevention of Recurrent Venous Thromboembolism clinical trial.^[^ ^114^ ^]^ Likewise, the cumulative distribution of the minimal lumen diameter was evaluated between two groups: carbon ion‐implanted and bare metal Arthos stents in the Asian Pacific Multicenter Arthos Stent Study trial. There was no statistical difference in two groups prior to the procedure, immediately following it, and at the 6‐month follow‐up.^[^ ^113^ ^]^ Furthermore, comparable results were also observed in preclinical investigation by Kapfer et al., where carbon‐coated (Carboflow) or uncoated expanded polytetrafluoroethylene (ePTFE) grafts were implanted femoral–anterior tibial artery bypass in 283 randomized patients.^[^ ^115^ ^]^

Despite the failure of PyC in preclinical studies, PyC has been considered as more biocompatible than many other materials and used in medical applications. Indeed, modern bileaflet mechanical heart valve components are still coated with carbon. Yet, people who get artificial heart valves necessitate to take systemic anticoagulants for the rest of their lives to prevent or reduce thromboembolic consequence. Traditional antithrombotic treatment has a number of drawbacks and restrictions, including oral administration discomfort, bleeding risks (from heparin aptamers), a limited therapeutic window, and unfavorable medication and food interactions (vitamin K antagonist‐warfarin) which way more seriously happens to novel anticoagulants.^[^ ^116^ ^]^ On the other hand, due to scratches at the valve hinge location, PyC components continue to malfunction and discharge carbon particles into the blood, potentially contaminating it.^[^ ^117^ ^]^

##### Diamond‐Like Carbon Coatings

Tetrahedral carbon (TaC) and diamond‐like carbon (DLC) films made of amorphous carbon have strong wear resistance, low friction coefficients, and chemical inertness, all of which contribute to good corrosion resistance.^[^ ^118^, ^119^ ^]^ As containing significant amounts of sp^3^ hybridized carbon atoms, DLC possesses typical properties of diamond, meanwhile TaC is regarded as the purer type of DLC with trace of sp^2^ hybridized bonded carbon. In DLC films, the hydrogen concentration varies by up to 40%, so usually designated by a‐C:H. Due to the amorphous structure, it can be embedded with other elements, such as N, F, Si, and metals, the characteristics of DLC and TaC may be further altered, making them more suitable for certain applications.^[^ ^120^ ^]^

In past efforts to enhance biocompatibility, films made of hydrogenated amorphous carbon have received the greatest attention. Investigations on the biocompatibility of amorphous carbon films created by ion beam deposition process were made in the early 1990s. By measuring the hemolysis ratio and observing platelet adhesion to its surface, the hemocompatibility of TaC was investigated. TaC was discovered to have superior anticoagulation properties to low‐temperature isotropic pyrolytic carbon, which is currently being used as the material for artificial heart valves in clinical use.^[^ ^121^ ^]^ The study of Logothetidis et al. first demonstrated that higher hydrogen concentration favors a higher albumin adsorption rather than fibrinogen, which ensues the inhibition of platelet adhesion.^[^ ^122^ ^]^ To compare with poly(methyl methacrylate) (PMMA), polyethylene (PE), polydimethylsiloxane (PDMS), medical steel, and TaC, Nurdin et al. prepared amorphous carbon coatings by plasma enhanced CVD approach and studied on three blood‐contacting events—coagulation, platelet activation, and inflammatory processes. The results showed that DLC tend to prevent activation of platelet and complement convertase. According to this study, the smoothness and inertness of the DLC film may be what accounts for its higher blood compatibility. Likewise, Ti‐dopped hydrogenated amorphous carbon gave similar results, which contributed to smoother topography.^[^ ^123^ ^]^ On the other hand, phosphorus incorporation lessened the protein interaction by increasing hydrophilicity with a WCA of 16.9° and preferring albumin adsorption.^[^ ^124^ ^]^ The investigation of Si‐ and F‐dopped DLC also resulted in the suppression of platelet attachment and activation. In which, the mechanism of Si incorporation underlies the lowering of interfacial tension and works as a semiconductor, whereas fluorinated carbon films improves the water‐shedding properties that turns out inhibiting protein adsorption.^[^ ^120^ ^]^ In respect of silicon carbide, despite the promising short‐ and long‐term outcomes, the restenosis rate, however, was similar to 316L stainless steel.^[^ ^125^ ^]^

Due to the outstanding thromboresistance in vitro compared to PyC, the impact of sp^3^ and sp^2^ hybrids was considered. As reported, the domination of sp^3^ portion resulted in improving hemocompatibility, and vice versa.^[^ ^122^ ^]^ These confusing conclusions revealed that hybridization was not objective enough to evaluate the antithrombosis properties. Further investigations were carried out to observe the influence of crystalline conformation in terms of hemocompatibility. According to Raman spectrometry measurement, the higher D‐band to G‐band intensity ratio showed improved blood compatibility in either hydrogenated or Si‐dopped DLC films.^[^ ^120^, ^126^ ^]^ In particular, given the multilayer architecture, a more intense D band is to some extent predicted, and signals are more disordered in the structure. This could explain for the irregularities in signals within molecule‐sized structure that elevates the free surface energy of the coatings and eventually encourages protein adsorption.

In vitro studies of macrophage interactions with DLC coatings have demonstrated less inflammatory responses and spreading than TaC.^[^ ^127^ ^]^ Similarly, different studies have confirmed the bioinertness of DLC that attributes for its biocompatibility. DLC films also proved the excellent corrosion resistance on metallic stents with no trace of metal release, meanwhile noncoated stents in contact with human plasma for 4 days have been shown to corrode.^[^ ^128^ ^]^ Trials of diamond‐like nanocomposite‐coated coronary stents were conducted in vivo, and the results matched those of the aforementioned in vitro studies. The stents were studied after 6 weeks being inserted into the coronary arteries of pigs. The results demonstrated that the DLC coatings were biocompatible and decreased thrombogenicity.^[^ ^129^ ^]^ In preclinical investigation, successful implantation of DLC‐coated stents was observed after 1 month and 6 months. No stent failure and thrombosis were found.^[^ ^130^ ^]^ Nevertheless, another clinical trial in 2004 found that coated stainless steel stents do not significantly outperform uncoated stents in terms of the binary restenosis and major adverse cardiac effects.^[^ ^131^ ^]^ Some applications of DLC coatings on guidewires reviewed the improvement in lubrication, stability, and hemocompatibility features in order to replace the conventional PTFE coatings.^[^ ^120^, ^132^ ^]^ Besides, co‐deposition of elements such as Si^[^ ^120^ ^]^ and F^[^ ^132^ ^]^ exhibited the enhanced lubrication, and adhesion to substrate as well as the decrease in thrombogenesis, delamination and spallation upon guidewire winding.

Overall, these studies showed that there is no consistent association between hemocompatibility and the carbon atomic bond structure or surface wettability. These characteristics do not seem to be the only ones influencing hemocompatibility. It should be noted that the precise mechanism of fibrinogen and albumin selective adsorption during blood‐biomaterial interaction is not completely known. The basic knowledge of how blood interacts with biomaterials might lead to a novel design for the hemocompatible surface. Furthermore, amorphous carbon still exists the mutual weakness of bulk material coatings that is substrate adhesion and spallation due to less flexibility, which limits its applications for blood‐contacting medical devices.^[^ ^120^ ^]^

##### Ultrananocrystalline Diamond Coatings

One of the most exciting and promising non‐polymeric anticoagulant coatings researched recently was the use of ultrananocrystalline diamond (UNCD). UNCD layer was introduced after conformally depositing a thin tungsten layer (20–100 nm). Thickness measurements indicated that the UNCD layer was roughly 1.5–2 µm thick. SEM images also depicted. It demonstrated an exceptionally smooth coating that has shown remarkable potential in terms of its anticoagulant capabilities, despite its challenge in manufacturing. In comparison with PyC, the contact with UNCD showed a less or comparable thrombin production level which still implies a small chance of blood clotting but in an acceptable level. The domination of sp3 hybridization resulted in improving hemocompatibility. Indeed, the hybridization of materials could strongly affect the surface energy and extensively have an impact on blood regulation, such as protein adsorption and platelet adsorption. Here UNCD is a sp^3^‐hybridised material that intrinsically possesses antithrombosis properties. In parallel, a much smaller grain cluster's size than the microstructure of PyC could further reduce the surface area as well as roughness. Furthermore, the UNCD integration minimized film stress or delamination up to a load of 100 N on a contact area of 3 × 10^−2^ mm which could be potentially applicable for mechanical heart valves.^[^ ^109^ ^]^

#### 4.1.3 Albumin Coatings

Albumin is a small globular and multifaceted protein which is abundantly circulating in human body.^[^ ^133^ ^]^ As reported, adsorbed albumin induces less non‐specific proteins adsorption compared to fibrinogen or globulin that turns out promoting less platelet adhesion and activation. Besides, due to its superior biodegradability, biocompatibility, nontoxicity, nonimmunogenicity, and water solubility, albumin is preferred over synthesized molecules in the biomedical area.^[^ ^134^ ^]^ Therefore, plasma albumin has been widely exploited for surface passivation as a stealth coating. The adsorption of albumin was proved to proceed by different mechanisms on either hydrophilic or hydrophobic surface, eventually resulting in an outer passivation layer(s) that could hinder further protein adsorption regardless surface composition.^[^ ^135^ ^]^ The denaturation of BSA followed by adsorption was not always observed.^[^ ^136^ ^]^ However, the competitive adsorption of plasma protein, which is a complex dynamic process relating to arbitrary sorption and desorption of different biomolecules, was not completely deciphered. Thus, the physical coatings of albumin have not been preferred for antithrombotic coatings.

As an endogenous biomolecule, coatings of albumin were often prepared by either self‐assembling or being covalently grafted on modified surfaces. Modifying substrate's nature beforehand can enhance the selective affinity for circulating albumin.^[^ ^137^ ^]^ Long aliphatic chains or warfarin have been tailored as an intermediate layer onto surfaces to encourage the binding of endogenous albumin. Specifically, most studies on long aliphatic chains concentrated on octadecyl isocyanate (C18) owing to its extreme hydrophobicity that favorably enables albumin absorption from bloodstream. In parallel, it was shown that the quantity of C18 rose with an increase in albumin adsorption.^[^ ^137^ ^]^ Various modification approaches of C18 cab were conducted, for instance, urethane linkage reaction, thiol‐ene “click chemistry,” or C18‐immobilised tetraethylene glycol‐terminated self‐assembled monolayers.^[^ ^138^, ^139^ ^]^ In general, C18‐modified surfaces produced a number of advantageous features, such as decreased complement activation, leukocyte adherence, and coagulation activity in vitro.^[^ ^140^ ^]^ Likewise, PEG‐tethered warfarin conjugates have been exploited due to an extremely strong binding between warfarin and albumin.^[^ ^141^ ^]^ However, albumin denaturation and enzymatic degradation were observed to occur in vivo on surfaces that have been permanently bound with albumin. Alternatively, covalently grafting of albumin has been widely utilized for surface functionalization. Albumin can be chemically conjugated; likewise, mussel‐inspired coatings based on polydopamine (PDA) have gained much interest, in which the PDA primary coating bound to amine groups on BSA via coupling with *o*‐benzoquinone moieties.^[^ ^142^ ^]^ These modified surfaces with albumin coating showed improved resistance to protein fouling and increased blood compatibility.

In 1976, the first glutaraldehyde‐crosslinked albumin coating was manufactured and applied to Dacron arterial grafts. As expected, the in vitro studies of Dacron displayed the decline in platelet and leukocyte adhesion as well as fibrin formation. For in vivo research, the albuminated and uncoated polyester grafts did not exhibit any significant increase of circulating T cells or T cell subsets, and neither did the circulating IL‐2 receptor‐positive cells for either graft type. Moreover, once albumin had fully resorbed, the healing behavior was comparable to that of the Vascular II prosthesis in terms of excellent tissue ingrowth and endothelialization.^[^ ^143^ ^]^ Another benefit of albumin‐coated grafts was flexibility and leakproof reliability with superior handling and suturing qualities. Notwithstanding promising results, studies in dogs (thoracoabdominal bypass model) and humans (prosthesis in the aortic position) failed to remarkably improve performance compared to uncoated implants.^[^ ^144^ ^]^

#### 4.1.4 Polymer Coatings

Coatings of hydrophilic, brush‐like polymers are often used to prevent protein adsorption and lessen interactions between proteins and blood cells on the foreign surface.^[^ ^145^ ^]^ Besides lowering surface energy, the hydration force and steric repulsion are the two dynamic processes that support this action. Strong repulsive hydration forces provide a stealth effect when a thin layer of water is securely bonded to a surface, preventing protein adsorption. The steric repulsion force, which is connected to compression and entropy reduction of hydrophilic polymeric chains immobilized on surfaces, makes up the second component.^[^ ^146^ ^]^ The density and molecular weight of the polymer utilized determine the repellent effect, and as the chain length of the polymer increases, protein adsorption on a functionalized surface decrease.^[^ ^147^ ^]^

##### Poly(ethylene glycol) Coatings

Poly(ethylene glycol) (PEG), poly(ethylene oxide) or polyoxyethylene is essentially the same polymer; however, it can be fabricated by using various monomers or polymerization processes, resulting in the structure repeat unit as (CH_2_─CH_2_─O)*_n_*. It was discovered in the early 1970s that PEG passively adsorbed onto glass surfaces inhibited the adsorption of thrombin, platelets, and viruses.^[^ ^148^ ^]^ PEG is a well‐known polymer that exhibits an extremely low adsorption of protein or cell.^[^ ^149^, ^150^, ^151^, ^152^, ^153^ ^]^ The high electronegativity of oxygen molecules along the polymer chains is thought to explain the high solvation of PEG, which turns out to be a liquid‐like surface and is thus not prone to biofouling.^[^ ^145^ ^]^ Besides, methylene and ethylene oxide groups are flexible groups that can easily exhibit conformational rearrangements. This nature makes PEG an impressive lubricant which complements the “washout effect” of PEG‐coating surfaces.^[^ ^154^ ^]^

According to the literature, the majority of PEG‐modified surfaces are impervious to protein and cell binding in vitro. For instance, PEG was grafted onto silicon substrates to demonstrate that the coating prevents BSA, IgG, and fibrinogen from adhering to the surface.^[^ ^155^ ^]^ Concurrently, Jin et al. used BSA and fibrinogen model to elucidate that high‐density PEG surfaces might significantly reduce protein adsorption and enhance biocompatibility.^[^ ^156^ ^]^ Principally, steric repulsion plays a vital role in PEG‐coated surfaces. Thus, the compression of surface‐bound hydrophilic PEG molecules reduces their mobility, which is energetically unfavorable and thermodynamically inhibits protein adsorption.^[^ ^6^ ^]^ As a consequence, the longer polymer chains immobilized, the lower density coated, therefore proteins may somehow penetrate and locate at adsorption sites. To overcome this problem, tetraglyme with low molecular weight PEG was synthesized by plasma grafting that exhibited the ultralow fibrinogen adsorption.^[^ ^157^ ^]^ Notwithstanding the well‐known acceptance for biomedical applications, it is found that PEG could activate the complement system and spark immunogenicity.^[^ ^158^, ^159^ ^]^ Moreover, PEG coatings may not last the lengthy lifespan in vivo due to oxidative degradation catalyzed by oxygen and transition metal ions which are inherently presenting in the physiological microenvironment.^[^ ^160^, ^161^ ^]^ Although the majority of PEGylated surfaces showed resistance to protein and cell binding in vitro, outcomes in vivo have been unreliable, and there is still no further investigation in preclinical.^[^ ^162^ ^]^

##### Other Polymer Coatings

A myriad of synthetic or natural polymers has been developed to increase hydrophilicity and biocompatibility. Van der Giessen et al. provided an informative picture about biodegradable polymers on five coronary stents in porcine model: poly(glycolic acid‐co‐lactic acid), polycaprolactone, poly(3‐hydroxybutyrate‐*co*‐3‐hydroxyvalerate), polyorthoester, poly(ethylene oxide‐*co*‐butylene terephthalate).^[^ ^163^ ^]^ Although these polymers have been widely reported in different medical applications, the team indicated that they still induced a significant inflammatory and proliferative response after 4 weeks. Furthermore, these polymeric compounds resulted in neointimal hyperplasia. On the other hand, non‐biodegradable polymers are also applied for antithrombotic coatings. For instance, linear polyurethane (PU) is distinguished by a distinctive urethane group (─NH─CO─O─). Polyurethane‐coated stents cause an inflammatory cell response that includes lymphocyte infiltration and a foreign‐body reaction, as well as the formation of multinucleated giant cells, according to animal experiments using the rabbit model. After 4 weeks, there has been no discernible impact on the rate of intima proliferation and the reduction of restenosis.^[^ ^164^ ^]^ Disappointing results were found in poly(ethylene terephthalate) (PET) and PDMS that evoked extensive inflammatory response with neointimal thickening and incident of restenosis,^[^ ^165^ ^]^ though PDMS had a potential in cellular proliferation that could encourage endothelialization. An exerting combination of poly(2‐methoxyethyl acrylate) (PMEA) and surface modifying additives have been demonstrated comparable results to heparin‐coated CPB.^[^ ^166^ ^]^ Yet, other investigations pointed out no perceptible benefit, such as relatively modest platelet count, transient leukopenia, elevated respiratory quotient, and C‐reactive protein levels, which might lead to post‐operative systemic inflammatory response syndrome.^[^ ^167^ ^]^ To conclude, it is not recommended to utilize these synthetic surfaces without additional anticoagulation.^[^ ^168^ ^]^

#### 4.1.5 Zwitterion Coatings

Zwitterionic molecules have demonstrated outstanding anti‐fouling properties, which have greatly increased their potential for anticoagulant coating.^[^ ^169^, ^170^, ^171^ ^]^ These molecules contain both positively and negatively charged groups, at physiological pH, the net charge is thus neutral.^[^ ^39^, ^172^, ^173^ ^]^ Zwitterion‐coated surfaces have a higher capacity for solvation through electrostatic interactions with water molecules compared to ordinary hydrophilic polymers, resulting in good protein resistance that is mostly attributable to repulsive hydration forces.^[^ ^174^, ^175^, ^176^ ^]^

##### Synthetic Zwitterion Coatings

Phosphobetaine, sulfobetaine, and carboxybetaine are the three typical zwitterionic residues that are most studied.^[^ ^177^, ^178^ ^]^ They have been grafted to the surfaces of titanium and titanium alloys to further improve the blood compatibility of these materials.^[^ ^179^, ^180^ ^]^ Likewise, polysulfobetaine‐functionalized catheters were designed by Smith et al. displayed a more than 98% decrease in platelet, lymphocyte, monocyte, and neutrophil activation when exposed to human blood. Even after catheters were exposed to serum in vitro for 60 days, the formation of thrombosis on the catheter surface was reduced by over 99% compared to unmodified control devices.^[^ ^178^ ^]^

Even though zwitterionic polymer coatings have shown potential results, their strong anti‐fouling properties are not helpful for some types of cell adhesion and growth.^[^ ^181^ ^]^ As mentioned above, the development of an endothelial layer on the surface of cardiovascular devices is an important factor in long‐term antithrombosis. Zwitterionic polymer is problematic for long‐term medical implants such as vascular grafts. The solution to this drawback is a zwitterionic polymer based on polysaccharides.^[^ ^182^ ^]^ In particular, this combination provides the benefit of the zwitterionic polymers to avoid thrombosis and resist non‐specific protein adsorption, and it also supplies the bioactive spot for the cells' particular adherence along polysaccharide chains.^[^ ^183^ ^]^ Indeed, Ye et al. engineered a series of polysaccharide‐based zwitterionic polymers via esterification reactions or atom transfer radical polymerization, which showed a strong potential for non‐specific protein resistance.^[^ ^184^, ^185^, ^186^ ^]^ To enhance the adhesive behaviors of cells, the RGD peptide, a cell‐specific adhesion polypeptide, can be alternatively bound on the polysaccharide‐based zwitterionic polymers.^[^ ^187^ ^]^

**Figure 3:**
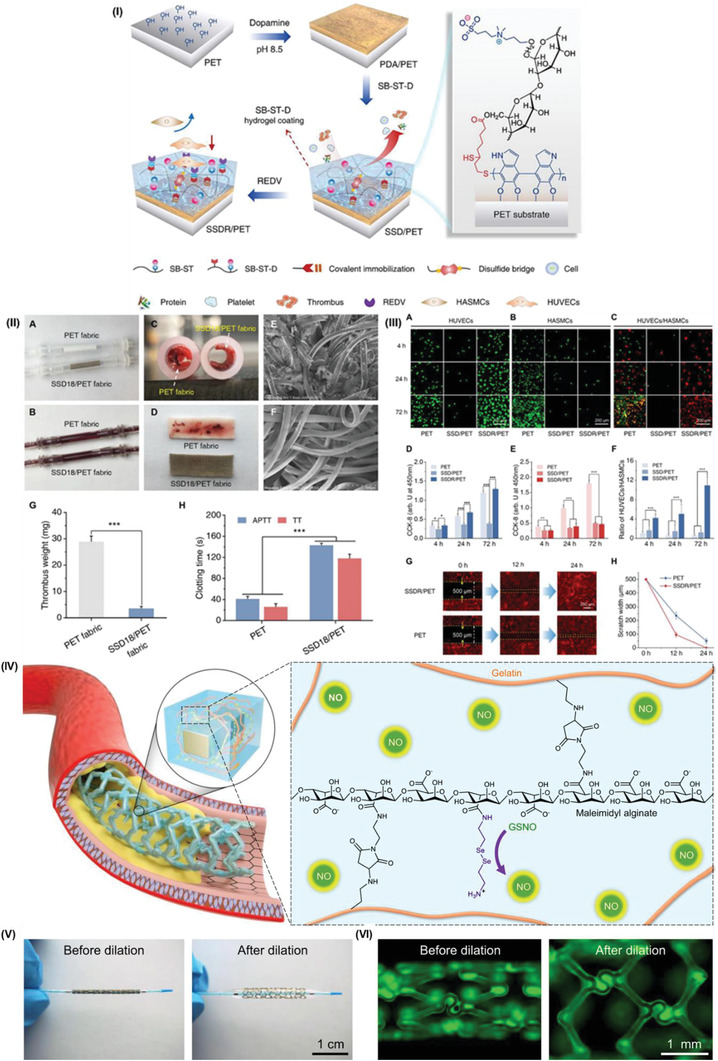
Starch‐based zwitterionic hydrogel coatings. I) Depiction of zwitterionic hydrogel coatings. II) Pristine PET fabric and SSD18/PET fabric A) before and B) after filling with blood, cross‐sectional and formed thrombus pictures of C,D) PET fabric and SSD18/PET fabric after exposed for 1 h, SEM images of E) pristine PET fabric and F) SSD18/PET fabric, G) thrombus weight determination, H) APTT and TT tests. III) Fluorescence images of competitive adhesion of A–C) HUVECs and HASMCs, proliferation of D) HUVECs and E) HASMCs within 72 h and F) their ratios grown on bare and coated surfaces, H) fluorescence images of scratch healing assay, and scratch width changes on PET and SSDR/PET surfaces at 0, 12, and 24 h (^*^ *p* < 0.05, ^**^ *p* < 0.01, ^***^ *p* < 0.001). Reproduced with permission.^[^ ^188^ ^]^ Copyright 2021, Elsevier. Another strategy that used gelatin and alginate for nitric oxide‐eluting (NOE) hydrogel coating on vascular stent to sustain nitric oxide release and suppress neointimal. IV) Schematic design of the NOE hydrogel coating. V) Macroscopic photos and VI) fluorescence images of the NOE hydrogel‐coated stent before and after dilation. Reproduced with permission.^[^ ^189^ ^]^ Copyright 2021, Springer Nature.

On the other hand, zwitterionic polymer coatings typically behave unpredictably in blood and body fluid microenvironments because of their extreme hydrophilicity and low mechanical stability.^[^ ^190^, ^191^, ^192^ ^]^ Although zwitterionic polymers could be immobilized on the device through the surface‐initiated atom transfer radical polymerization,^[^ ^193^ ^]^ they are fragile and decomposable due to oxygen, shear stress, and transition metal ions catalysis.^[^ ^194^ ^]^ Several efforts have been conducted. For instance, coating stability can be increased by combining hydrophobic subdivisions with zwitterionic polymers.^[^ ^195^ ^]^ Nevertheless, the hydrophobic polymer will undoubtedly compromise the coating's overall anti‐fouling performance. Most recently, the invention of zwitterionic hydrogel coatings can surmount with all above issues as follows:^[^ ^196^, ^197^, ^198^, ^199^, ^200^ ^]^ 1) the hydrogel maintains the antifouling nature of zwitterionic molecules, 2) it can bond to the substrate through various interactions (covalent bond, ionic bond, and hydrogen bond, etc.), 3) cross‐linking network within hydrogel structure can sustain the stability of the coating, 4) zwitterionic hydrogels can incorporate with other factors, such as bioactive molecules, polymers, nanoparticles to obtain desired purposes.^[^ ^201^ ^]^ For instance, a critical effort of Yao et al. has shown incredible performance in anti‐clotting and competitive adhesion of endothelial cells against smooth muscle cells (**Figure** **3**I,II,III).^[^ ^188^ ^]^ Another attempt from Yao et al. was zwitterionic microgel‐based coating, which is bio‐inspired by fish skin and vascular endothelial layer, offering a controllable and high stability up to 14 days.^[^ ^202^ ^]^ Altogether, though zwitterionic polymer‐modified surfaces demonstrated higher resistance to protein and cell binding as compared to PEGylated surfaces, in vivo and in clinical results have not been carried out so far.

##### Phosphorylcholine Coatings

Phosphorylcholine (PC), which is a derivative of phosphatidylcholine⁠—the major glycerophospholipid presents in animal cell membranes has been proven to inhibit protein and cell adhesion in vitro. PC is a bio‐mimicking zwitterionic molecule.^[^ ^163^ ^]^ Due to the intrinsic nature of zwitterion, PC is electrically neutral at physiological pH, carrying both positive and negative charges. In addition, phospholipid‐mimetic characteristic gives an advantage in cell‐to‐cell communication through changes in charge and composition.^[^ ^203^ ^]^

Among a myriad of PC derivatives, 2‐methacryloyloxyethyl phosphorylcholine (MPC) was widely employed by numerous approaches. For example, individual MPC molecules can form a monolayer or bilayer film via weak hydrophobic or van der Waal interactions, which obviously limits by the inherent instability.^[^ ^204^ ^]^ Similarly, the disulfide‐modified PC was self‐assembled by the high affinity of gold substrate.^[^ ^205^ ^]^ Other routes could be accounted for the incorporation of MPC groups alongside the polymethacrylate and polyurethane‐based polymers or the in situ polymerization of MPC into biomimetic film, which proposed more stable and stealth coatings.^[^ ^206^ ^]^ Generally, the exposed PC layer maintained its protein‐ and cell‐resistant qualities throughout all the trials. Concurrently, some in vivo experiments also resulted in excellent blood compatibility, significantly reduced platelet deposition, and limited neointimal hyperplasia.^[^ ^207^ ^]^ Nonetheless, phosphorylcholine polymer‐coated stents did not outperform uncoated stents in pig or rabbit angioplasty models.^[^ ^208^, ^209^ ^]^ Although several PC‐based coatings were commercialized, no data are available to support usage without ongoing systemic anticoagulation.^[^ ^210^ ^]^

#### 4.1.6 Elastin‐Inspired Coatings

Elastin is a structural protein found in the vascular wall which causes only moderate platelet adhesion and aggregation. In the late 1990s, Ito et al. first applied tissue‐derived elastin to Dacron vascular grafts and observed smooth muscle cell migration inhibition without affecting ECs’ motility, which suggested that such a coating would prevent the development of anastomotic intimal hyperplasia.^[^ ^211^ ^]^ Although these conventional coatings employed elastin derived from bovine or pig tissues, elastin is still exerted as a challenge for separating and purifying due to low solubility. The discovery of an elastin consensus sequence that served as the foundation for the manufacture of protein polymers inspired by elastin provided a solution to this issue.^[^ ^212^, ^213^ ^]^ Hereafter, soluble and elastin‐inspired oligopeptide poly(Val‐Pro‐Gly‐Val‐Gly) was synthesized in lieu of nature‐derived. It is reported that the coating reduced fibrinogen and immunoglobulin adsorption in vitro as well as less release of proinflammatory cytokines by monocytes.^[^ ^214^ ^]^ Another study allowed elastin polypeptide (EP20‐24‐24) passively adsorbed on three synthetic materials: PET (Mylar), poly(tetrafluoroethylene‐co‐ethylene) (Tefzel), and polycarbonate with PU (Corethane). The in vitro experiments using platelet rich plasma showed that all three synthetic materials inhibited platelet activation and adhesion. When compared to uncoated controls, polyurethane catheters coated with the polypeptide demonstrated a large improvement in catheter patency and a significant decrease in fibrin accumulation and embolism in a rabbit model.^[^ ^215^ ^]^ Furthermore, elastin‐mimetic protein polymer films also diminished the thrombogenicity in an ex vivo shunt model.^[^ ^216^ ^]^ Shun model is an ex vivo model where a vessel loop was inserted from artery to vein. The occlusion of blood on the coated loop will be assessed after given time. To conclude, elastin‐inspired polymers are potential candidates for antithrombotic surface coating, despite the fact that there is no further development in preclinical investigation and the majority of present efforts are concentrated on using them for targeted therapeutic drug delivery and tissue adhesives.^[^ ^217^, ^218^ ^]^

#### 4.1.7 Textured and Omniphobic Coatings

Another method to increase the hemocompatibility of blood‐contacting biomaterials is to model the surface topography to produce textured surfaces (e.g., by tailoring geometric features of surfaces with voids or fibrils). In respect of textured surfaces, the hemocompatibility results are expected from the way textured surfaces encourage the establishment of a solid biological lining, known as a pseudo neointimal layer composed of adsorbed and denatured proteins and other entrapped blood plasma components.^[^ ^219^ ^]^ A number of techniques can be used to engineer the surface topography and produce textured surfaces, such as using sintered titanium or argon plasma etching to prepare micropatterns on titanium oxide layers, or solvent casting of PU by using molds of patterned cavities, and particle casting to create geometric features on surfaces.^[^ ^220^ ^]^ In vivo results revealed the neointimal development on textured surface and decreased thrombosis formation as compared to non‐textured one.^[^ ^221^ ^]^ Although these approaches were found to be risky and uncontrollable because the topography of the surface has a significant impact on the development of a stable neointimal layer on the material, several Ti‐based products have been commercialized for the ventricular assist devices applications.^[^ ^222^ ^]^

**Figure 4:**
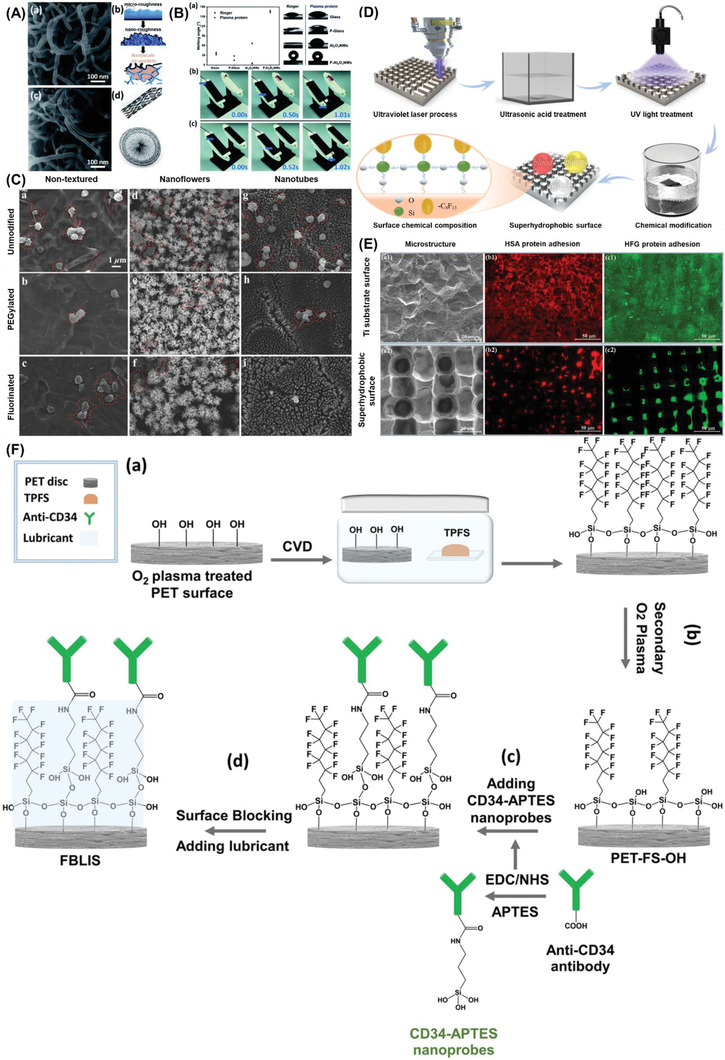
PTFEP–Al_2_O_3_ hybrid nanowires preventing biofouling and thrombosis. A) Helium ion microscopy images of a) pristine Al_2_O_3_ nanowires and c) PTFEP–Al_2_O_3_ nanowires, schematic illustrations of b) the surface morphology of Al_2_O_3_ nanowires, and d) photos of PTFEP–Al_2_O_3_ nanowires coated cardiovascular implants. B) Contact angle analysis and cross‐sectional images of a) the corresponding droplets. Comparison of b) blood drop sliding on (video‐captured images) PTFEP–Al_2_O_3_ nanowires versus the glass (control) substrate and c) PTFEP–Al_2_O_3_ nanowires versus pristine Al_2_O_3_ nanowires. Reproduced with permission.^[^ ^223^ ^]^ Copyright 2019, Royal Society of Chemistry. C) SEM images revealed platelet adhesion and activation on different titanium‐based substrates. Reproduced with permission.^[^ ^224^ ^]^ Copyright 2017, John Wiley and Sons Ltd. D) Fabrication process of superhydrophobic titanium plates. E) Magnified image of the microtexture and fluorescent microscope images of adsorbed albumin and fibrinogen on different surfaces. Reproduced with permission.^[^ ^225^ ^]^ Copyright 2021, Elsevier. F) Schematic illustration of preparing lubricant‐infused PET surfaces using silanized CD34–APTES nanoprobes. Reproduced with permission.^[^ ^226^ ^]^ Copyright 2019, Wiley‐VCH GmbH.

A novel surface modification has been gaining a lot of interests because it virtually repels any sorts of lipid, so‐called omniphobic surface. One of the first attempts was inspired by the *Nepenthes* pitcher plant that creates a low friction surface using a layer of liquid water to stop insects from attaching. Leslie et al. invented heparin‐free coating by using a tethered‐liquid perfluorocarbon (TLP) surface including a tethered perfluorocarbon layer and a liquid perfluorocarbon on top of that.^[^ ^227^ ^]^ This TLP coating is the first surface coating to lessen thrombosis in vivo without using anticoagulants, and with a modest level of anticoagulation (0.25 U mL^−1^ heparin) in ex vivo peristaltic pumping model. Technically, by significantly lowering a solid surface's free surface energy at the three‐phase contact point to increase its hydrophobicity, a superhemophobic surface is created, following the classical Young equation.^[^ ^228^ ^]^ The contact angle referred to superhydrophobic surface is more than 150°. At this wettability, superhydrophobic surfaces offer a supremely slippery layer which is different from ordinary hydrophobic surfaces. Fluorination has thus been used the most in this regard because of its innate hydrophobicity and biocompatibility in both preclinical and clinical.^[^ ^229^ ^]^ For instance, poly(bis(2,2,2‐trifluoroethoxy)phosphazene) (PTFEP) has been coated on Al_2_O_3_ nanowires and significantly reduced the in vitro platelet adherence.^[^ ^223^ ^]^ Besides, air pockets at nano‐roughness nanowires are thought to contribute to the “slipping over” effect of the surface against plasma protein and platelet, as illustrated in **Figure** **4**.^[^ ^230^ ^]^ Concurrently, other textured surfaces (TiO_2_ nanoflowers/nanotubes, UV laser treated Ti surface) have been covered by fluorinated moieties to assess the effect of superhydrophobicity on antithombogenicity.^[^ ^224^, ^225^ ^]^ Also, lubricant‐infused surfaces were able to suppress biofouling and restrain occlusive thrombosis. However, due to their excellent antibiofouling performance, cell repellence is unavoidable which limits the process of re‐establishing a functional endothelial cell layer on the surface of the implant, leading to less integration and long‐term stability.^[^ ^231^ ^]^ In order to enhance targeted binding of biomolecules, built‐in anti‐CD34 antibody was incorporated upon the lubricant layer to promote targeted binding of endothelial cells on top of the superhydrophobic surface and attenuate thrombin generation.^[^ ^226^ ^]^ Nevertheless, the durability of lubricant under in vivo conditions is questionable, as they must withstand the shear forces of blood flow and maintain their efficacy over time. There is a possible risk that the coating could degrade or wear off under harsh conditions. To summarize, fluorinated or lubricant‐infused surfaces are emerging with its excellent antibiofouling characteristic; however, there is still room to investigate their usability in various vascular implants or practical medical devices.

### 4.2 Inhibition of Thrombin Generation and Fibrin Formation

**Figure 5:**
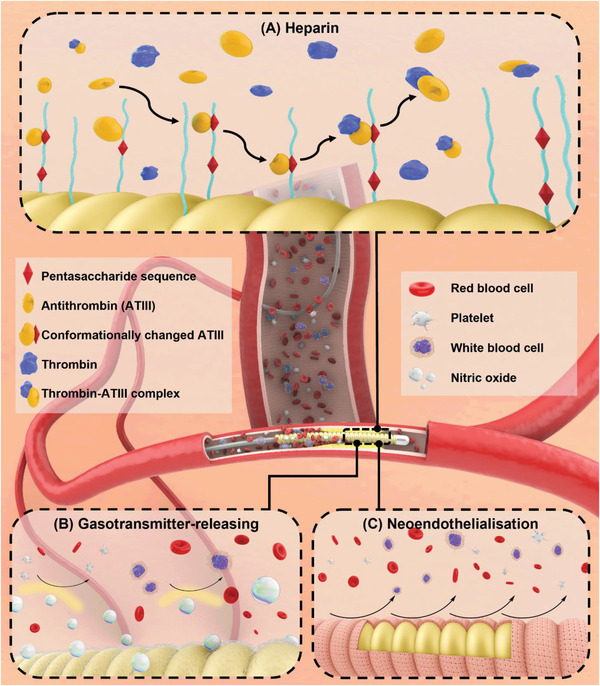
Illustration of several bioactive coatings, describing mechanisms of A) action of heparin, B) gasotransmitter‐releasing, and C) endothelialization toward the inhibition of coagulation factors.

Endothelial cells display and release several physiologically active components to reduce thrombotic reactions. ECs seeding or other forms of reconstitution on a prosthetic surface have been intensively studied to create thromboresistant vascular replacements. However, this method has its own difficulties that have been described elsewhere regarding cell source, stability, viability, and function. In general, a variety of anticoagulant medications are recommended for use as hemocompatible alterations to medical equipment. Coagulation factors are the primary targets of the many anticoagulants, which include direct and indirect coagulation inhibitors, anticoagulant proteins, and contact system‐specific inhibitors. The use of bioactive chemicals such as heparin, thrombomodulin, or urokinase on the surface of synthetic materials has also shown encouraging outcomes through biologically inspired or biomimetic designs (**Figure** **5**). This approach specifically imitates the antithrombogenic properties of the ECs layer. A summary of materials that inhibit thrombin and fibrin formation was listed in **Table** **5**.

**Table 5:** Critical studies in surface coatings inhibiting thrombin generation and fibrin formation for antithrombosis.

Substrate/device	Coating	Coating technique	Critical properties		Key results	Ref.
PU/catheter	ATIII‐heparin‐PEG	ATIII‐heparin complex and PEG covalently bonded to the catheter surface	—	—	Attracted ATIII in the presence of plasma proteins by heparinized surfaces Showed lower binding of the fibrinogen and fibrin degradation products Revealed the antithrombotic behavior by anti‐fXa activity	[272]
Polyacrylonitrile/hemodialysis membrane	Polyethylenimine‐heparin	Polyethylenimine ionically layered onto polyacrylonitrile membrane and bound to UFH	—	—	Allows a 50% reduction of standard doses of UFH administration without increasing the risk of massive clotting Remained in platelets, hemoglobin levels, erythropoietin needs, and dialyzer performances	[273]
Platinum/amperometric glucose sensor	PA‐PEG‐heparin	PA‐PEG‐heparin physically coated on platinum wire	—	—	Suggested the suppression of blood coagulation by a high resistance to platelet adhesion	[274]
PHEMA–albumin hydrogel	Low molecular weight heparin	Low molecular weight heparin covalently bound to hydrogel via ester linkages	*D* *θ*_c_ FIB	78.6 µg cm^−2^ 45.1° <3 µg cm^−2^	Reduced protein adsorption and platelet adhesion Antifouling properties regarding to fibrinogen, and reduction in cell adhesion in vitro	[275]
PU	Heparin‐DA	DA‐conjugated heparin attached to PU substrate	*t* *θ*_c_	26.46 ± 4.1 Å 62.0°	Mussel‐inspired coating showed to be sufficient Improved blood compatibility, preventing blood coagulation and platelet adhesion Remained the anticoagulant activity for 7 days in vitro	[276]
PVC/sodium selective electrode	Chitosan and chitosan‐heparin	Chitosan and chitosan‐heparin physically attached to PVC film	—	—	Chitosan‐heparin modified film indicated less thrombogenicity with sheep platelet rich plasma in vitro	[277]
Co─Cr stent	Heparin and four‐armed PEG hydrogel	Hydrogel solution sprayed by air‐brush device	*R*_a_	<128.6 nm	Suppressed the stent‐induced coagulation activation without a major increase of the global heparin concentration	[278]
PES	Albumin‐heparin (standard and high affinity) multilayer	Adsorbed albumin‐heparin multilayers fixed by glutaraldehyde	*D*	1.23‐0.155 µg cm^−2^ (standard) 1.26–0.200 µg cm^−2^ (high affinity) 1.06 µg cm^−2^ (BSA only)	Reduced the platelet either adhesion or activation, and leukocyte adhesion Relatively low complement activation	[279]
PES	Sulfated alginate	Dopamine‐conjugated sulphated alginate self‐assembled on the PES substrate	*θ*_c_ *R*_a_ BSA FIB	<46.1 ± 2.3° ≈23 nm <8 µg cm^−2^ <7.5 µg cm^−2^	Maintained excellent anticoagulant activity in vitro Promoted cell growth and proliferation Exhibited relatively low platelet adhesion/activation and complement activation	[280]
Gold‐coated silicon wafer	PEG (≈1100 Da)‐hirudin	PEG‐hirudin covalently bonded to gold via sequential and direct methods	*t* *θ*_c_ FIB	27 ± 8 Å (sequential) 31 ± 9 Å (direct) ≈35° (sequential and direct) 20–35 ng/cm^2^	Showed less protein resistance compared to PEG‐alone surface and highly interaction to thrombin Enhanced thrombin inhibitory activity by the immobilized hirudin	[281]
Gold	PEG (≈1100 Da)‐CTI (sequential and direct method)	PEG‐CTI covalently bonded to gold via gold‐thiol chemistry	*t* *θ*_c_ FIB	23 ± 5 Å (sequential) 21 ± 9 Å (direct) ≈34° (sequential) ≈49° (direct) <0.4 µg cm^−2^ (buffer) <0.05 µg cm^−2^ (50% plasma)	Lessened the fibrinogen adsorption in the multi‐protein environment of plasma in vitro Improved the anti‐fXIIa bioactivity by CTI moieties, thereby prolonging clotting time	[282]
PEBAX and PTFE/PCI guide catheter, or PU/single lumen catheter	PEG (3500 Da)‐CTI and PEG‐ovalbumin	PEG conjugates self‐assembled on the substrates	*θ*_c_ fXII FIB	77.5 ± 5.4° (PEG‐CTI) 73.6 ± 4.9° (PEG‐ovalbumin) ≈2 ng cm^−2^ <40 ng cm^−2^	Reduced the fXII and fibrinogen adsorption in vitro Expressed low fXIIa activity and less production of fXIa in CTI‐modified catheter Prolonged the occlusion time in CTI‐coated catheter in vivo	[271]
PLA	Hirudin	Hirudin bound to poly(vinylpyrrolidone‐*co*‐vinyltriethoxysilane) via hydrogen bonding	—	—	Showed excellent thromboresistance even over 12 h incubation time The higher hirudin immobilization the higher anti‐clotting property Lowered platelet adhesion and the activated C3a and C5a concentrations	[283]

PU—polyurethane, UFH—unfractionated heparin, PES—polyethersulfone, PCI—percutaneous coronary intervention.

#### 4.2.1 Glycosaminoglycan‐Based Coatings

##### Heparin Coatings

Heparin was used for the first time therapeutically in the late 1930s to treat deep vein thrombosis. According to Gott et al., the immobilization of heparin on the surfaces of biomaterials dates back to the early 1960s and has been considered the most efficient coating method.^[^ ^248^ ^]^ Heparin modifications are frequently employed to improve the hemocompatibility of biomaterials and have found utility in a variety of medical devices, including dialysis membranes, vascular stents, and ventricular assist devices.^[^ ^8^, ^249^ ^]^ The anticoagulant effect of heparin is brought on by its indirect inhibition of thrombin, fXa, as well as fIXa and fVIIa on a less frequent basis. In particular, the primary physiological inhibitor of thrombin⁠—antithrombin (ATIII)⁠—is a circulating glycoprotein of hepatic origin that is not dependent on vitamin K. The anticoagulant action of ATIII is at least 1000 times more potent in the presence of glycosaminoglycan, particularly heparin. Heparin functions as a coagulation inhibitor underlying a catalytic mechanism which makes it theoretically “renewable.” The mechanisms by which heparin activates ATIII is as depicted in Figure 5A. First, ATIII binds to a sequence‐specific pentasaccharide site on heparin chains which induces conformational change in the serpin. Then, the conformationally changed ATIII hastens the inactivation of thrombin or other coagulation factors by promoting a ternary complex formation. Finally, the complex is released to the blood stream and the heparin molecule is once again exposed, maintaining the anticoagulant activity. Heparin has therefore been proven to be the most efficient cofactor for thrombin inhibition.^[^ ^250^, ^251^ ^]^ Most importantly, thrombin—ATIII complexes necessitate active binding sites along heparin molecules, thus heparin molecules with fewer than 18 saccharides are not sufficient to inhibit thrombin generation. Moreover, heparin has anti‐inflammatory characteristics in addition to its anticoagulant action by preventing the complement system and leukocyte activation.^[^ ^252^ ^]^

**Figure 6:**
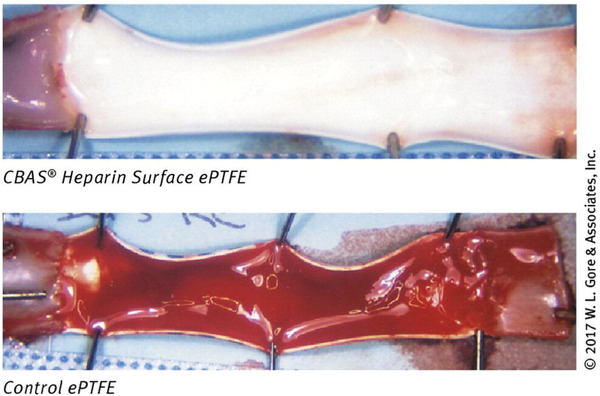
Macroscopic photographs of the lumen of an ePTFE vascular graft containing the CARMEDA BioActive Surface Heparin Surface compared to an uncoated control after 2 h in a challenging non‐anticoagulated canine carotid interpositional model. The coated surface eliminated thrombotic occlusion, decreased inflammatory response, infection rates, and neointimal hyperplasia. Reproduced with permission.^[^ ^249^ ^]^ Copyright 2017, Elsevier.

Heparin can be immobilized onto artificial surfaces by various strategies,^[^ ^248^, ^250^ ^]^ where electrostatic interaction and covalent attachment are the most prevalent approaches. With regard to the anticoagulant action of heparin, the manner of immobilization is crucial.^[^ ^253^ ^]^ Principally, heparin as a highly negatively charged biopolymer can be electrostatically adsorbed on the positively modified surfaces (e.g., amine groups). However, the physically bound heparin is prone to exchange by other ionic substances and leach into the bloodstream under shear stress. Hence, the covalent conjugation of heparin on coating surfaces was established to overcome those weaknesses. With an abundance of functionalities along the polymer chains, the covalent linkages can be created for immobilization by following two main pathways: side‐on and end‐on. In a side‐on manner, heparin is conjugated via covalent bonds along its structural backbone, which could lessen the density of anticoagulant and the availability of targeted sites, thereby reducing the catalytic capacity of heparin.^[^ ^190^, ^254^, ^255^ ^]^ Here, the difficulty with heparin coatings is that for each heparin polymer chain to function, ATIII serine protease inhibitors must first attach to heparin to generate the heparin‐ATIII complex.^[^ ^253^, ^256^, ^257^ ^]^ In a realistic scenario, this situation could be trivialized due to limited reactive sites along the polysaccharide backbone which are influenced by the cover density, attachment routes, and steric hindrance. By contrast, end‐point attachment highly deliberates the polymer chains and in turn, boosts the pentasaccharide sequence content that can encourage the generation of the heparin‐ATIII complex. Carmeda AB company has successfully translated its BioActive Surface technology that employed the end‐on grafting of heparin with retained bioactivity to actively prevent platelet adhesion and thrombus formation on medical device surfaces (**Figure** **6**).^[^ ^248^, ^253^, ^258^ ^]^ Technically, each heparin chain consists of a reducing hemiacetal at one end which is in equilibrium with a ring‐opened state containing a deprotected aldehyde and will readily react to strong nucleophiles, such as amine groups.^[^ ^259^ ^]^ Therefore, one end of each heparin chain can be attached to an amine‐bearing surface to improve heparin capability. Furthermore, another drawback of heparin is that it eventually loses the capacity to combine with ATIII owing to deterioration brought on by oxidative factors from surrounding biological environment.^[^ ^258^ ^]^

Despite the fact that there are several ways to immobilize heparin to a substrate, only a small number of technologies have been effectively used in commercially available medical devices, and only a small portion of these technologies have non‐eluting surfaces as its primary design. Even fewer of these commercial covalent chemistries have concrete proof of surface heparin bioactivity. The quantity of public material that is accessible, especially of a technical nature, varies greatly among the commercial technologies, as well as the amount of data has not been found in the published literature due to confidentiality, making a thorough comparison between them so far difficult.

##### Heparin‐Mimicking Coatings

As discussed above, one of the most widely utilized biopolymers for enhancing surface blood compatibility and anticoagulant action is heparin. Also, heparin‐coated surfaces were found to increase hydrophilicity, cell growth activity, and simultaneously inflammatory responses. Nevertheless, due to long‐term exposure to biological systems and interactions with blood components, the use of heparinized surfaces may result in a significant loss of their bioactivity in vivo.^[^ ^260^ ^]^ Moreover, the price and availability of heparin may limit itself from heparin‐immobilized surface coating applications. Porcine mucosa and bovine lungs are the main sources in the heparin industry.^[^ ^261^ ^]^ Thus, it is necessary to offer alternative polymers that can simulate the role of bioactive heparin which are bioavailable and cost‐effective.

Ionic functional groups like sulphate, sulfamide, and carboxylate groups are claimed to be the primary contributors to the anticoagulant action of heparin.^[^ ^262^ ^]^ Therefore, numerous efforts have been placed in designing heparin‐like macromolecules to mimic the structure of heparin by tailoring sulfonic acid groups and/or carboxylic acid groups, which are motivated by the chemical structures of heparin. Through a variety of facile chemical techniques, heparin‐like or ‐mimicking polymers might be easily manufactured on a massive scale. Additionally, the generated heparin‐like polymer‐modified membranes have demonstrated exceptional blood and cell compatibilities, that suit heparin‐immobilized membranes.

In one of the first investigations on heparin‐like polymers, Pereira et al. created sulfated fucans, which were made of oligosaccharide repeating units with sulfation, to imitate the anticoagulant of heparin.^[^ ^263^ ^]^ In contrast to mammalian glycosaminoglycans, the linear fucans from echinoderms often needed the presence of ATIII or heparin cofactor II for the indirect inhibition of thrombin. The branching fucans from brown algae were shown to be direct inhibitors of thrombin. Another naturally occurring heparin‐like or ‐mimicking substance was identified from crabs by Andrade et al. Its structure was rich in disulfated disaccharides and contained a lot of 2‐O‐sulfated‐beta‐d‐glucuronic acid units.^[^ ^264^ ^]^ Although this heparin‐like/heparin‐mimicking molecule only demonstrated a minimal in vitro anticoagulant activity and low bleeding potency, it could become a promising candidate for the creation of structure‐driven, heparin‐based therapeutic medicines with few side effects.

Altogether, heparin‐mimicking polymers brought positive outcomes for the alternatives of heparin. However, the underlying mechanism may vary. It can base on the binding effect of pentasaccharide sequence, which is analogous to pristine heparin. It can also rely on the brush‐like polymer manner when heparin‐like polymers act as highly negatively charged biopolymer. Moreover, recent developments in heparin‐mimicking polymers can provide more structurally defined compounds that can focus on a single biological interaction, such as anticoagulant activity exclusively. Alike semi‐synthetic polysaccharides, some synthetic classes of sulfonated polymers can also increment blood coagulation time and minimize platelet adhesion with increasing sulfonation incorporation, such as polycaprolactone, poly(glutamic acid), poly(methyl methacrylate‐*co*‐styrene sulfonate), etc. Otherwise, some sulfonation modifications on specific synthetic polymers can further offer promotion or inhibition of FGF2‐induced cell proliferation in endothelial cells. Although desulfation of sulfates still probably happen in vivo, sulfated synthetic polymers have the benefit of being stable when compared to semi‐synthetic or heparin itself.^[^ ^184^ ^]^ To conclude, this class of materials proposes not only alternative approaches for heparin usage in antithrombotic treatment but also desirable purposes of sulfonated polymers targeting to certain biofunctions.

#### 4.2.2 Thrombin Inhibitors Coatings

Anticoagulant proteins have been fixed to the biomaterial surfaces of vascular stents or vascular grafts, including thrombomodulin,^[^ ^265^ ^]^ activated protein C,^[^ ^266^ ^]^ or tissue factor pathway inhibitor.^[^ ^267^ ^]^ In protein C anticoagulation pathway, protein C is activated by thrombin. The presence of thrombomodulin and endothelial protein C receptors significantly aids the activation of protein C. For instance, thrombomodulin has been covalently attached to nitinol surfaces that have been functionalized by amino terminated organosilanes.^[^ ^268^ ^]^ While a small number of platelets still bound to the surface, the capacity to promote protein C activation still presented. However, there were numerous drawbacks, including deterioration in vivo, activity loss upon sterilization, and high cost.^[^ ^269^ ^]^ Besides, covalent immobilization of thrombin inhibitors may hinder the thrombin binding and lower their bioactivity.^[^ ^266^, ^270^ ^]^

Corn trypsin inhibitor (CTI) was utilized as a surface modification to bind to and inhibit fXIIa. CTI is a well‐known selective and powerful inhibitor of fXIIa with a 12 500 Da protein produced from maize kernels.^[^ ^271^ ^]^ Since CTI inhibits fXIIa by forming a 1:1 complex, it should be understood that the amount of CTI present on a surface will determine how much inhibition is possible. Studies have shown that the surface modified with CTI displays reduced fibrinogen adsorption and inhibits fXIIa in vitro. In preliminary in vivo experiments using PEG‐CTI catheters, promising results were obtained, but further research is necessary before they can be used in medical practice.^[^ ^271^ ^]^ However, it is important to note that CTI may also inhibit downstream coagulation factors, such as thrombin and platelets, indirectly impacting the contact pathway activation.

### 4.3 Inhibition of Platelet Aggregation and Activation

Another modification strategy for preventing thrombus development on blood‐contacting medical equipment is the use of blood platelet inhibitors. In this method, the antiplatelet agents can be attached on surface or eluted from a coating matrix which will target circulating platelet via distinct mechanism to regulate the aggregation and activation. There are various platelet agonists that have been used for investigational coatings such as prostaglandin E1, dipyridamole, apyrase, GPIIb/IIIa inhibitor, honokiol, nitric oxide, hydrogen sulfide, etc.

#### 4.3.1 Platelet Inhibitors Coatings

Prostaglandin E1 (PGE1) has been preferred as a medication in antithrombotic therapy. Since 1990s, methods to prevent platelet activation and aggregation on biomaterials have exploited the use of lipid PGE1.^[^ ^284^ ^]^ The immobilization of PGE1 has shown relatively promising output.^[^ ^285^ ^]^ In particular, PGE1 acts as a potent vasodilator of all arterioles which stimulates adenyl cyclase activity in platelets and elevates cyclic adenosine monophosphate (cAMP) levels, thereby prohibits Ca^2+^ release and platelet aggregation brought on by P2Y1 receptor activation.^[^ ^284^ ^]^ Additionally, an increase in albumin surface attachment and a decrease in fibrinogen binding were seen in the PGE1‐immobilised substrate.^[^ ^286^ ^]^ As the strongest platelet activators, ATP and ADP are also desirable targets to inhibit the activation of platelet. Hence, apyrases, a class of enzymes that possesses the unifying ability to catalyze the sequential hydrolysis of ATP to ADP and ADP to cAMP releasing inorganic phosphate.^[^ ^284^ ^]^ On polystyrene surfaces containing immobilized apyrase, reduced platelet activity was observed.^[^ ^287^ ^]^ Similarly, numerous antiplatelet drugs with analogous mechanism of action have been used for surface coatings. According to in vitro investigations, reduced thrombogenicity was linked to decreased platelet adhesion.^[^ ^284^ ^]^ Recently, a new generation of antiplatelet has attracted a great deal of interest—honokiol, which is a multifunctional bioactive natural substance and widely known for its great biological qualities, including those inhibiting platelet activation and aggregation caused by collagen and further have anti‐inflammatory, antibacterial, and antioxidant effects. The immobilization of honokiol revealed an outstanding outcome in either thromboresistance or other properties in vivo.^[^ ^288^, ^289^ ^]^

#### 4.3.2 Gasotransmitter‐Releasing Coatings

In the vasculature, the healthy ECs synthesize a gaseous signaling molecule—nitric oxide (NO) via nitric oxide synthase catalysis from the l‐arginine substrate, approximately 0.5–4 × 10^−10^ mol cm^−2^ min^−1^. NO is the endogenous mediator that contributes to the endothelium's tonic atheroprotective, thromboresistant, and vasodilator effects by affecting the activity of circulating cells and underlying smooth muscle.^[^ ^290^ ^]^ Besides, NO is also an inflammatory mediator that could regulate the expression of pro‐inflammatory factors and deactivate monocytes by promoting macrophage polarization from the inactivated phenotype to the anti‐inflammatory phenotype.^[^ ^291^ ^]^ Hence, the bio‐inspired incorporation of NO for antithrombotic coatings is the most promising strategy that can suppress the reciprocal relationship between thrombosis and inflammation, promote endothelialization, and inhibit smooth muscle cell proliferation, as illustrated in Figure 5B.^[^ ^58^, ^292^, ^293^, ^294^, ^295^ ^]^ More specifically, numerous platelet deactivation activities, including as shape change, secretion, and calcium signaling, are impacted by NO. NO interacts with a variety of target proteins in platelets mostly via activating soluble guanylyl cyclase via the cyclic guanosine monophosphate (cGMP) pathway and then cGMP‐dependent protein kinase, which accounts for the diversity of its inhibitory function.^[^ ^296^ ^]^ **Table** **6** summarizes several novel coatings that have been reported.

**Table 6:** Critical studies in surface coatings inhibiting platelet aggregation and activation, and promoting neoendothelialization.

Substrate/device	Coating	Coating technique	Critical properties		Key results	Ref.
PVC and silicon	Honokiol	Honokiol coated on amine‐rich substrate	*R*_a_	63.0 nm	Inhibited collagen‐induced platelet activation and aggregation, and excellent antithrombotic properties in vitro, in vivo Inhibited the activation, growth, and secretion of inflammatory factors (TNF‐α and IL‐6) in macrophages	[288]
Silicone rubber/amperometric oxygen‐sensing catheter	NO‐releasing diazeniumdiolates	Diazeniumdiolate‐doped cocktail solution dip‐coated with silicone rubber tubing	—	—	Prevented thrombogenicity on the implanted sensors in vivo due to the absence of platelet adhesion	[325]
CarboSil	SNAP and zwitterionic copolymer	SNAP incorporated in the substrate and coated by zwitterionic copolymer	*t* *θ*_c_	≈50 nm ≈50°	Resisted moderate abrasion for over one week with retention of its antifouling property Sustained release of NO up to 2 weeks	[298]
Si and 316L stainless steel	TiO*_x_*, CuO*_y_*	High‐power pulsed magnetron sputtering/ DC magnetron sputtering co‐sputtering	*t* *θ*_c_	>180 nm >80°	Promoted the viability and proliferation of ECs, suppressed the viability of SMCs Inhibited the platelet adhesion and activation	[326]
PHB and PLA fibers	GSNO	Fibrinogenres electrospun using solution of PHB, PLA, and GSNO	*—*	—	Reduced platelet adhesion by 64.6% Proven to be effective cellular adhesion and activity platforms without harm to mammalian cells	[327]
PVC	HA/DTris@Cu	Coatings chemically bound to amine‐rich surface	*θ*_c_ FIB	<40° ≈1 a.u. (control) ≈0.5 a.u.	Demonstrated dual synergistic effects, including antifouling function of hyaluronic acid and the signaling effect of NO releasing Showed minimal platelet adhesion, fibrinogen resistance, antimicrobial properties in vitro, and prevented thrombogenesis ex vivo	[299]
PVC	CuNPs‐immobilized by heparin‐TA	Cross‐linked heparin‐tyramine and entrapped CuNPs immobilized on PVC via hydrogen bonds	*t* *θ*_c_	8.07 nm 45°	Generated NO in situ that inhibited the adhesion and proliferation of platelets and SMCs, and enhanced the migration of ECs in vitro Prevented blood clotting in vitro Reduced the inflammatory response by improving M2 macrophage polarization	[328]
PET vial	SNAP/PDA/heparin	SNAP‐impregnated PET coated by PDA‐heparin	—	—	NO/heparin surface modification reduced platelet aggregation and surface thrombus development with human whole blood Showed heparin eroding from the immobilized surfaces	[329]
316L stainless steel	H_2_S donor/chitosan/HA	H_2_S donors loaded in self‐assembly layers of catechol‐modified chitosan and HA	*θ*_c_	<70°	Inhibited platelet adhesion and activation, fibrinogen adsorption, and denaturation Prevented the proliferation of SMCs and reduced the inflammatory response Encouraged the formation of new blood vessels Decreased thrombogenesis ex vivo blood circulation	[330]
Ni─Ti	VEGF and anti‐CD34 antibody	Dip‐coated the PDA/heparin‐coated substrates in the solution of VEGF and anti‐CD34	*θ*_c_ BSA	<40° ≈900 µg cm^−2^ (control) <100 µg cm^−2^	Significantly reduced the protein adsorption Captured EPCs and promoted the proliferation and migration of HUVECs Provided better biocompatibility than the bare stent	[331]

PVC—poly(vinyl chloride), PHB—polyhydroxybutyrate, PDA—polydopamine, EPC—endothelial progenitor cell.

**Figure 7:**
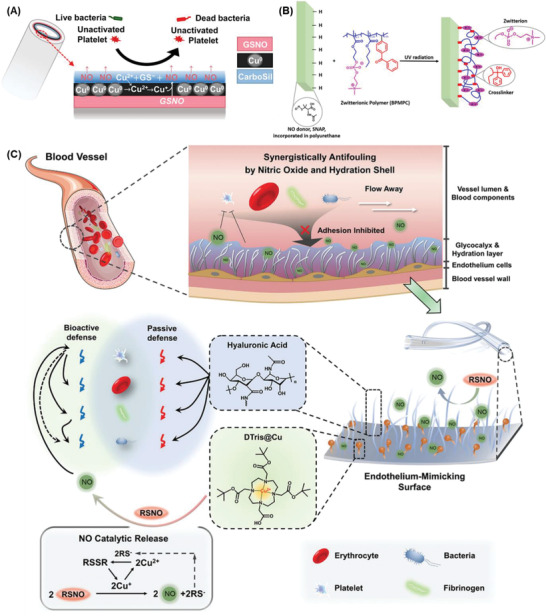
Endothelium‐mimicking surface combining both biopassive and/or bioactive defenses to combat thrombosis and biofouling. A) The multilayer coatings consisted of endogenous donor S‐nitrosoglutathione (GSNO), copper nanoparticle (Cu^0^), and CarboSil layers. Reproduced with permission.^[^ ^297^ ^]^ Copyright 2019, American Chemical Society. B) The NO donor (S‐nitroso‐*N*‐acetylpenicillamine, SNAP) was embedded into CarboSil coating and covalently grafted with antifouling zwitterionic terpolymer (2‐methacryloyloxyethyl phosphorylcholine‐*co*‐butyl methacrylate‐co‐benzophenone, BPMPC). Reproduced with permission.^[^ ^298^ ^]^ Copyright 2017, American Chemical Society. C) The PVC tubing with hyaluronic acid (HA) and tri‐tert‐butyl 1,4,7,10‐tetraazacyclododecane‐1,4,7,10‐tetraacetate‐chelated Cu^2+^ (DTris@Cu) coatings. Reproduced with permission.^[^ ^299^ ^]^ Copyright 2021, Wiley‐VCH GmbH.

Regarding the local NO‐releasing strategies, there are two main pathways either directly or by catalysis. The first method is a direct release technique by immobilizing NO donors, such as N‐diazeniumdiolates (NONOates), metal/NO complexes, nitrates, nitrites, N‐nitroso, C‐nitroso, S‐nitroso.^[^ ^300^ ^]^ In general, NO can inhibit platelet aggregation in vitro and in vivo.^[^ ^301^ ^]^ Although these substances are thought of as NO donors, they exhibit various pharmacokinetic and dynamic characteristics that determine the intensity of their biological effects. The overproduction of NO and vice versa are correlated with high blood pressure and numerous human diseases or side effects.^[^ ^302^ ^]^ S‐nitroso‐N‐acetylpenacillamine (SNAP) and NONOates are often used for NO‐releasing research, for instance, a 4‐h extracorporeal circulation in rabbits revealed the antithrombogenic properties when embedded (SNAP) and diazeniumdiolated dibutylhexanediamine into PVC tubing.^[^ ^300^, ^303^ ^]^ Similarly, SNAP‐doped Elast‐eo E2As released the mild NO flux that reduced the thrombogenicity by 90% as compared to controls in sheep model for 7 days.^[^ ^304^ ^]^ Novel NO donors or NO catalysts has been developing to improve the sustained release of NO and brought up promising outcome, as shown in **Figure** **7**.^[^ ^299^ ^]^ However, only a finite extent of NO donors may be integrated into the polymer reservoir, which poses a restriction on the utilization of NO‐releasing components. Recently, research indicated that extended NO‐release for longer than 30 days could be possible.^[^ ^304^, ^305^, ^306^ ^]^

Catalysts utilizing physiological sources such as S‐nitrosothiols (RSNOs) for NO synthesis is an additional choice to compensate the limitation of NO‐doped coatings. Transition metal ions and their organic derivatives are often exploited to catalyze NO production in different cascades, including copper, zinc, selenium, iron.^[^ ^307^, ^308^ ^]^ Obviously, the NO production highly depends on the endogenous NO concentration. Some first attempts immobilized organoselenium species and sustained catalytic activity in vitro over 30 days.^[^ ^309^ ^]^ Besides, catalysts could be impregnated within the metal‐organic frameworks (MOFs).^[^ ^310^, ^311^, ^312^ ^]^ or incorporated as nanoparticles.^[^ ^297^, ^313^, ^314^ ^]^ This class of materials is still lacking compelling in vivo evidence. Despite the fact that this method can prolong the local NO generation, the endogenous RSNO amount may not be sufficient when there is no supplemental RSNO administered. Therefore, this is a major barrier for NO‐generating materials since they cannot simulate the physiological flux as well as the fluctuation of RSNO concentrations in different individuals.^[^ ^315^ ^]^ Although zinc element at trace concentration is appreciable for maintaining healthy ECs structure and function, the significant leakage of heavy metal ions could accumulate and harm organs.^[^ ^308^ ^]^

As another anti‐inflammatory and vasoactive gasotransmitter, hydrogen sulfide (H_2_S) was speculated to serve as a physiologically inhibitory signal.^[^ ^316^ ^]^ Indeed, H_2_S has exhibited the inhibition of platelet aggregation by acting as a wide range of agonists against ADP, collagen, and thrombin.^[^ ^317^ ^]^ In H_2_S‐releasing coatings, the facile incorporation of Na_2_S has been often employed as the most prevalent approach. It is reasonable to hypothesize that H_2_S plays an inhibitory effect comparable to that of NO. Zhong et al. found that a variety of collagen‐induced signaling events were decreased thereby aggregation was inhibited, along with secretion and calcium mobilization from intracellular reserves when the H_2_S concentration was from 100 µm to 10 mm.^[^ ^318^ ^]^ On the contrary, H_2_S‐producing salts have been shown to increase human platelet aggregation triggered by thrombin receptor activator peptide (TRAP‐6) and to contribute to the increased platelet aggregation seen with hyperhomocysteinemia at lower doses (0.1–100 m).^[^ ^319^ ^]^ Therefore, further study is warranted to investigate the rationale for inhibitory and stimulatory effects of H_2_S upon regulating platelet activation and aggregation.

### 4.4 Neoendothelialization

Only the inner surface of blood arteries, or vascular endothelium, may actually be non‐thrombotic. Natural antithrombotic features of endothelial cells include the existence and release of the anticoagulant heparan sulphate, thrombomodulin, and NO. To emulate the natural anti‐thrombotic inner lining of blood vessels, recreating or simulating the endothelium layer would be a potential strategy to boost the hemocompatibility of blood‐contacting medical devices. In this way, endothelial coatings could possibly regulate all destined thrombogenic stimuli such as protein adsorption, thrombin, and platelet.

From this point of view, in vitro cell seeding is one engineering technique for the endothelialization of material surfaces (Figure 5C). To clarify, this method focusses on the pre‐seeding procedure prior to blood‐contacting implantation. This method differs from the aforementioned approaches to promote cell adhesion by modifying cell‐selective peptides and releasing NO. Here, autologous, or allogenic ECs are extracted, cultivated, and then seeded on the blood‐contacting device surface prior to implantation.^[^ ^320^, ^321^ ^]^ However, due to issues with cell sourcing, cell stability, and cell viability, in vitro ECs seeding is a difficult operation that takes a lot of time and resources.^[^ ^270^ ^]^ Additionally, the requirement for “two‐pot” operations when using autologous cells raises the risk of contamination and infection. The use of allogenic cells might also result in rejection.^[^ ^322^ ^]^

As a consequence, several attempts have been made. In vivo self‐endothelialization brought on by endothelial progenitor cells may be a potential technique for imposing those restrictions.^[^ ^320^, ^322^ ^]^ Principally, the method relies on immobilizing antibodies, DNA or peptide aptamers, or anti‐cadherin on the surface of the substance in order to collect endothelial progenitor cells from a patient's circulating blood. This enables their attachment and subsequent differentiation, which in turn promotes the formation of an EC layer on the surface that comes into contact with blood. However, because this technique is not extremely selective, other cells may potentially and competitively proliferate on the same surface.^[^ ^323^ ^]^ Anti‐proliferative medication modifications have been proposed for the devices to prevent restenosis (growth of vascular smooth muscle cells) on the biomaterial.^[^ ^324^ ^]^

## 5 Physicochemical Characteristics of Coatings Influencing the Antithrombosis of Biomaterials

As discussed above, there is a myriad of surface coatings and biomaterials that provide the antithrombotic feature. However, there are no common trends in characteristics among diverse materials. Changes in any physicochemical properties of antithrombotic coating, such as thickness, topography, crystallinity, surface charge, or surface energy, might be correlated with various modes of interaction and result in various impacts on antithrombotic responses. Moreover, these factors proportionally affect the thromboresistance in a complex mechanism of action. Thus, it would not be able to conclude the most impactful stimulus in this circumstance.

### 5.1 Influence of Surface Topography and Roughness

Protein adsorption and the ensuing cell response are both significantly impacted by surface shape. It is indicated that proteins bind to nanostructures, but it is challenging to draw broad conclusions about the degree of adhesion and whether the proteins would get denaturized on these surfaces. Several studies revealed that conformational change in proteins irreversibly occurs when the surface roughness is remarkably minor or greater than the dimension of proteins. This happens less in the surface roughness that has the same order as protein size.^[^ ^332^ ^]^ Also, nanotopography interrupts the surface area and wettability, resulting in the overall effect of surface toward protein adsorption.^[^ ^333^ ^]^ Nanopore size has a massive impact on complement activation as well. Antibodies and complement proteins are prone to adsorb on large nanopore size, leading to the higher chance of triggering inflammation.^[^ ^334^, ^335^ ^]^ On the other hand, different nanotopographies were constructed to achieve high aspect ratio features of the surfaces, such as grooves, pillars, bumps, nanotubes, or nanowires.^[^ ^336^ ^]^ High aspect ratio features refer to structures or patterns on a surface that have a large height or depth compared to their lateral dimensions. In other words, the structures are “tall” and have a small width or diameter. These topographies can positively influence antithrombotic properties in several ways. For example, regularly spaced ridges and grooves can affect the orientation of platelets and prevent their activation.^[^ ^337^ ^]^ Likewise, other textures, for example, nanowires, nanopillars were hypothesized to create a physical barrier that can prevent platelet to adhere to and reduce the likelihood of thrombus formation.^[^ ^223^ ^]^ However, the size or dimension of topographies plays an important role for the textured surfaces, they can result in regulating the binding of platelets or adversely inducing the binding and activation. In particular, 100 nm groove size revealed a reduced platelet activation in comparison with 500 nm.^[^ ^338^ ^]^

The behavior of cells on various nanostructured surfaces has been the subject of intensive research. As a fragile component, thrombocyte is readily to be fractured and magnifies the platelet aggregation and activation.^[^ ^339^ ^]^ Cells may detect the chemistry and morphology of the surface they cling on. Cell filopodia (0.25–0.5 µm wide and 2–10 µm long), which contact with the surface and form focal adhesions. Also, filopodia can interact with the surface with features ranging from µm to nm, either randomly or geometrically structured. Nanoscale (1–500 nm) structures can trigger a particular cell response beyond micrometers. Additionally, it was well recognized that cell type and other factors affect how cells recognize shape. In parallel, surface topography also plays a vital role in providing three‐dimensionality to cell attachment.^[^ ^340^, ^341^ ^]^ Therefore, it could be able to induce a particular biological response in cells by appropriately modifying surface topography at the micro‐ and nanometer scales. This would be a very appealing method of creating biomaterial surfaces for certain uses, specifically endothelialization strategy.

### 5.2 Influence of Wettability and Surface Energy

One of the most significant factors that strongly affect how biological components respond to biomaterials is surface wettability.^[^ ^342^ ^]^ As mentioned, macromolecular proteins adsorb to the surface as soon as biomaterials interfere with biological fluids, and those compounds will influence later biological reactions between surface and blood stream. However, prior to plasma proteins, water molecules are considered the first ones, on nanosecond time scale, to reach the surface. Since water interacts and binds differently depending on the surface characteristics, proteins and other molecules, which arrive a bit later, can adhere to the surface to a certain extent and be contingent upon the as‐formed water shell. Next, the exchange processes between various proteins are further influenced by the intensity of protein interactions, and certain exchanges may not even occur.^[^ ^43^, ^46^, ^343^ ^]^ Cell–surface interactions are ultimately interactions between cells and surface–bound proteins (or other biomolecules) because cells primarily react to a protein‐covered surface when they reach the surface. To conclude, wettability has a significant impact on the quantity and structural changes of proteins that are adsorbed as well as platelet and cell adhesion.^[^ ^344^, ^345^ ^]^

To exploit the influence of wettability on regulating blood coagulation, surface free energy is often engineered. As a rule of thumb, increment of surface energy quickly develops a water shell and compromises an anti‐biofouling property in the early stage of biomedical devices when it contacts to blood stream. On the other hand, hydrophobic surfaces with minimal free energy prevents molecular attraction themselves. It should be noted that moderate hydrophilicity or hydrophobicity cannot make up a sufficient effect to eliminate biofouling. In fact, most research has focused on fabricate a hydrophilic surface with a modest wettability. This preference reveals that wettability and/or surface energy were only considered a secondary factor for passive defense. The breakthrough development of superhemophobic surfaces within the last five years has set a new foundation for anti‐clotting mechanism by repelling all kinds of biomolecules and become inherently inert in blood stream.

Overall, although it is likely that surface wettability contributes significantly to a material's ability to withstand blood flow, no direct link has yet been established between surface wettability and blood compatibility because the findings of several research are still debatable. This is likely caused by the complicated chemical pathways in blood, as well as the interaction of other significant surface characteristics, such as surface chemistry, morphology, and charge.

### 5.3 Influence of Functional Groups and Surface Charge

The chemical composition of surface and the biological reaction with blood are tightly related. The addition of surficial functional groups, such as hydroxyl (─OH), amine (─NH_2_), methyl (─CH_3_), or carboxylic (─COOH), is one method to increase the surface's hemocompatibility. This is either used to modify the biological response (allow immobilization of biomolecules, increase cell proliferation, decrease platelet adhesion, etc.) or both (enzymes, proteins, etc.).

Numerous studies have been done on how functional groups affect hemocompatibility, however the results have been somewhat controversial and unreliable. For example, enhanced surface hydroxyl concentration often leads in increasing complement activation. Increased methylation generally results in decreased complement activation. However, methyl‐decorated surfaces could somehow promote plasma protein binding.^[^ ^346^, ^347^ ^]^ Intriguingly, the research by Wilson et al. has demonstrated that ammonia and nitrogen plasma treatment of polymer (PU) surface (incorporation of nitrogen groups) greatly lowered contact activation.^[^ ^348^ ^]^ However, after oxygen and argon plasma treatment, there were no differences in thrombogenicity as compared to the untreated surface (incorporation of oxygen groups).

Another method modifying surface features by enriching the surface with functional groups and promoting cell growth is oxygen or nitrogen plasma treatment.^[^ ^349^ ^]^ Furthermore, the early adsorption of surrounding biological fluid containing diverse ions could result in the characteristics of coatings. For instance, phosphate ions were known to rapidly bind to titanium surfaces. These ions can alter the kinetics and structure of BSA adsorption on TiO_2_.^[^ ^350^ ^]^ This is either used to modify the biological response (allow immobilization of biomolecules, increase cell proliferation, decrease platelet adhesion, etc.) or both (enzymes, proteins, etc.).

### 5.4 Influence of Other Characteristics

Regarding inorganic materials, coatings are highly dependent on crystalline structure. Different arrangements at molecular level result in distinct surface energy that inherently affects the interaction of biomaterials–blood plasma. In particular, due to the well‐organized structure of PyC, coated surfaces have low surface energy, which prevents proteins and cells from adhering to them.^[^ ^351^ ^]^ On the other hand, flaws and imperfections in molecular structure will raise the free surface energy and subsequently promote the adsorption of proteins.^[^ ^120^, ^126^ ^]^

Likewise, the density of organic materials also impacts the anti‐biofouling effect and the coagulation cascade. For example, proteins may penetrate the coating and aggregate at adsorption sites if the polymer chains are too long and the density of the coating is low due to steric hindrance. In parallel, the effectiveness of bioactive coatings as anti‐clotting agents depends on the presence of specific active sites on the coating material. A major challenge in surface modification technology is to maintain the antithrombosis function of coatings by preserving the medicine or bioactive sites on surfaces. To clarify, densifying bioactive sites or remaining biofunction after coating is the advanced pathway to overcome conventional coatings’ shortcomings. An example of a successful approach is the use of CARMEDA BioActive Surface technology, which uses proprietary end‐point attached heparin that retains its bioactivity.^[^ ^352^ ^]^ This approach allows a higher density of bioactive sites of heparin chains, resulting in a significant improvement on antithrombotic properties. Similar to the honokiol coating mentioned above, only the attachment via the ortho position of the phenolic hydroxyl groups of honokiol can efficiently introduce the drug to a surface and reserve its biological functions.^[^ ^288^ ^]^

## 6 Clinical Challenges and Future Perspectives

Blood‐contacting surfaces/devices are the focus of biomaterial research to completely prevent the possibility of thrombus development. The advancement of material science leads to a myriad of novel materials that are more resistant to protein and cell adsorption. The consequent activation of platelets, coagulation, and complement has been used for antithrombotic applications. Although the fundamental mechanisms of these materials are relatively understood, there is still room for development in terms of materials and processing methods. Although there were numerous reports on antithrombotic coating materials, only a few classes of coatings can actually be translated to commercial coating products and applied in a real scenario in human body. Typically, commercial coating strategies employed diamond‐like carbon, phosphorylcholine‐based zwitterion, textured surfaces, heparin, and endothelialization. Most products are based on heparin and/or zwitterionic coatings because of their availability, ease in fabrication, and high efficiency in antithrombosis. As shown in previous sections, other materials exhibit excellent performance in in vivo studies; however, due to the unmet needs for safe, prolonged, and effective prevention of surface‐induced thrombosis on blood‐contacting surfaces/devices, more research is required to address a number of aspects for effective and safe thromboresistant materials:

1. According to ISO 10993–4 guidelines, most antithrombotic coatings assessment should take five distinct endpoints into consideration: thrombosis, coagulation, platelets, hematology (hemolysis and leukocyte count), immunology (complement activation).^[^ ^353^ ^]^ Excluding those devices that are not implanted in the circulatory system (or short duration on contact), a straightforward in vitro hemolysis test can be performed. Apparently, in vivo research is highly recommended. The behavior of antithrombotic coatings is not always predictable and reliable under physiological settings due to distinct blood conditions and characteristics. It might be impacted by shear stress, blood components (platelet, RBC, leukocyte concentrations), etc. These traits must be taken into consideration to simulate the actual coatings’ performance in further investigations.
2. Future research is still needed since not all antithrombotic materials have been thoroughly studied. Their underlying mechanisms of action are not completely understood. Increasing number of novel coatings has been constantly reported. It is encouraged to establish the systemic investigation of each material influence on thromboresistance.
3. Numerous coatings were engineered with polymeric materials. Most of these polymers are biodegradable over time. This characteristic will limit the long‐term applications of polymeric coatings. Therefore, the influence of biological fluid on the degradation of coating layers needs to be carefully considered.
4. As mentioned, we suggest that the inflammatory response from innate immune system may cause or result from thrombosis.^[^ ^354^ ^]^ Hence, the trigger of the inflammatory cascade should be taken into account besides coagulation cascade. We recommend that the activation of leukocyte or complement system by anticoagulation surface need to be confirmed.
5. Since clotting cascade is a downstream pathway involving various coagulation factors, one interference at any point or factor might lead to a demise of thrombosis formation. Currently, only a limited number of coagulation factors have been targeted, such as thrombin (fIIa), platelet, fXa, fXIIa, and fIXa, fVIIa on a less frequency. Yet, there is still room for more discoveries on other anticoagulation factors that could efficiently inhibit the surface‐induced thrombosis.
6. Most studies in antithrombotic coatings often utilize one sort of material; however, commercialized products combine various materials, resulting in a wide‐range impact on blood‐clotting regulation. Apart from coatings that only target to a certain factor in the clotting cascade, coatings have a wide range of influence (NO, H_2_S, ECs, honokiol, etc.) may be the future for blood‐contacting device coatings.^[^ ^317^, ^355^ ^]^ Those materials not only disable multiple anticoagulation factors but also possess various beneficial properties, such as anti‐inflammatory, antibacterial, which could be exploited for a multifunctional surface instead of a combination of plenty of materials.

## 7 Conclusion

Blood‐material interactions that cause coagulation and thrombus still impose a difficulty for the utilization of blood‐contacting devices. The present review provides a full picture of the coagulation cascade, surface modification methods, and potential regulation by utilizing various coating materials. By categorizing coatings according to mechanism of action, we elaborated the influence of each material to specific targets that could contribute to the inhibition of surface‐induced thrombosis. Besides, the impact of coatings on the innate immune system, neoendothelialization which may benefit long‐term applications of medical devices, is also discussed. These summary findings could open new research avenues into blood coagulation and contact activation on biomaterial surfaces that include platelets, proteins, and surfaces. The systematic study of biological reactions to surfaces will be extremely helpful for developing biomaterials with better biocompatibility and further hasten their successful translation to the clinic and market.

## Conflict of Interest

The authors declare no conflict of interest.
